# Association of caesarean delivery with offspring health outcomes in full-cohort versus sibling-comparison studies: a comparative meta-analysis and simulation study

**DOI:** 10.1186/s12916-023-03030-2

**Published:** 2023-09-08

**Authors:** Hong-zhao Yu, Xiao-wei Wang, Zhen-yu Guo, Zhi Lin, Yu-bo Zhou, Hong-tian Li, Jian-meng Liu

**Affiliations:** 1 Institute of Reproductive and Child Health, National Health Commission Key Laboratory of Reproductive Health, Peking University Health Science Center, Beijing, China; 2 Department of Epidemiology and Biostatistics, School of Public Health, Peking University Health Science Center, Beijing, China; 3 Center for Intelligent Public Health, Institute for Artificial Intelligence, Peking University, Beijing, China

**Keywords:** Caesarean delivery, Offspring health outcomes, Cohort, Sibling comparison, Systemic review, Meta-analysis, Simulation

## Abstract

**Background:**

Full-cohort and sibling-comparison designs have yielded inconsistent results about the impacts of caesarean delivery on offspring health outcomes, with the effect estimates from the latter being more likely directed towards the null value. We hypothesized that the seemingly conservative results obtained from the sibling-comparison design might be attributed to inadequate adjustment for non-shared confounders between siblings, particularly maternal age at delivery.

**Methods:**

A systematic review and meta-analysis was first conducted. PubMed, Embase, and the Web of Science were searched from database inception to April 6, 2022. Included studies (1) examined the association of caesarean delivery, whether elective or emergency, with offspring health outcomes; (2) simultaneously conducted full-cohort and sibling-comparison analyses; and (3) reported adjusted effect estimates with 95% confidence intervals (95% CIs). No language restrictions were applied. Data were extracted by 2 reviewers independently. Three-level meta-analytic models were used to calculate the pooled odds ratios (ORs) and 95% CIs for caesarean versus vaginal delivery on multiple offspring health outcomes separately for full-cohort and sibling-comparison designs. Subgroup analyses were performed based on the method of adjustment for maternal age at delivery. A simulation study was then conducted. The simulated datasets were generated with some key parameters derived from the meta-analysis.

**Results:**

Eighteen studies involving 21,854,828 individuals were included. The outcomes assessed included mental and behavioral disorders; endocrine, nutritional and metabolic diseases; asthma; cardiorespiratory fitness; and multiple sclerosis. The overall pooled OR for estimates from the full-cohort design was 1.14 (95% CI: 1.11 to 1.17), higher than that for estimates from the sibling-comparison design (OR = 1.08; 95% CI: 1.02 to 1.14). Stratified analyses showed that estimates from the sibling-comparison design varied considerably across studies using different methods to adjust for maternal age at delivery in multivariate analyses, while those from the full-cohort design were rather stable: in studies that did not adjust maternal age at delivery, the pooled OR of full-cohort *vs.* sibling-comparison design was 1.10 (95% CI: 0.99 to 1.22) *vs.* 1.06 (95% CI: 0.85 to 1.31), in studies adjusting it as a categorical variable, 1.15 (95% CI: 1.11 to 1.19) *vs.* 1.07 (95% CI: 1.00 to 1.15), and in studies adjusting it as a continuous variable, 1.12 (95% CI: 1.05 to 1.19) *vs.* 1.12 (95% CI: 0.98 to 1.29). The severe underestimation bias related to the inadequate adjustment of maternal age at delivery in sibling-comparison analyses was fully replicated in the simulation study.

**Conclusions:**

Sibling-comparison analyses may underestimate the association of caesarean delivery with multiple offspring health outcomes due to inadequate adjustment of non-shared confounders, such as maternal age at delivery. Thus, we should be cautious when interpreting the seemingly conservative results of sibling-comparison analyses in delivery-related studies.

**Supplementary Information:**

The online version contains supplementary material available at 10.1186/s12916-023-03030-2

## Background

Caesarean delivery plays a crucial role in tackling medical conditions, such as abnormal placentation, dystocia, fetal distress, and previous caesarean delivery [1]. Over the past 5 decades, the global caesarean delivery rate has increased from 5% in 1970 to 21.1% in 2018 [2], exceeding the level of 15% endorsed by WHO [3]. The growing popularity of caesarean delivery has caused widespread concern about its potential negative impacts on maternal and offspring health [4]. Population-based cohort studies from different settings suggest an association of caesarean delivery with multiple health outcomes in offspring, such as obesity, asthma, type 1 diabetes, and attention deficit hyperactivity disorder (ADHD) [5–9], but whether these findings reveal causation has remained much debated primarily due to potential biases from uncontrollable confounders. More recently, studies have attempted to sidestep such confounding effects by using a sibling-comparison design, which could presumably adjust for unmeasured confounding factors shared by siblings (e.g., cultural background, parental characteristics, and child-rearing practices) and thus may generate more reliable results in some contexts [10, 11]. In most studies that simultaneously used these two designs, the sibling-comparison analyses did generate less significant results with respect to the impacts of caesarean delivery on offspring health outcomes, enhancing the speculation that the associations observed in full-cohort analyses were likely due to uncontrolled or residual confounding [12–16]. However, whether sibling-comparison analyses are more reliable than full-cohort analyses in this specific context remains largely unknown.

Mathematically, effect estimates from studies with sibling-comparison versus unpaired full-cohort design may be more biased due to the confounding of non-shared factors among siblings [17]. Maternal age at delivery may be an important non-shared confounder in delivery-related studies using a sibling-comparison design. Specifically, in these studies, only sibling pairs that differ in delivery mode will be informative on the estimated associations. Given that caesarean delivery after a previous vaginal birth is more frequent than vaginal birth after a previous caesarean (VBAC) [18–20], the artificial selection of siblings with different delivery modes would lead to a systematic upwards bias in the maternal age for caesarean-born compared to vaginally-born siblings, as compared with a full-cohort design. In the meanwhile, higher maternal age might be associated with lower risks of adverse health outcomes of offspring, as older mothers generally have higher socioeconomic status and better parenting experience [21]. This indicates that maternal age at delivery, as a confounding factor, may counteractively reduce the negative impacts of caesarean delivery on offspring health outcomes. Therefore, we raised the hypothesis that sibling-comparison studies, compared with full-cohort studies, would be more likely to underestimate the true association of caesarean delivery with offspring health outcomes due to inadequate adjustment for maternal age at delivery.

In this study, we first performed a systematic review and comparative meta-analysis for studies using both full-cohort and sibling-comparison designs to investigate the association between all caesarean delivery, including both elective and emergency caesarean delivery, and offspring health outcomes, with a particular focus on the impacts of different handling methods of adjustment for maternal age at delivery in multivariate regression models. We then conducted a simulation study to explore whether the results of the meta-analysis could be replicated mathematically.

## Methods

This systematic review and meta-analysis was conducted and reported according to the Preferred Reporting Items for Systematic Reviews and Meta-Analyses (PRISMA) guidelines [22].

### Search strategy and eligibility

We initially searched PubMed, Embase, and the Web of Science on November 4, 2020, and updated the search on April 6, 2022. We combined terms related to “caesarean delivery”, “cohort study”, and “siblings comparison design” without restrictions on language and health outcomes. Full details of the search strategy are provided in Additional file 1. We also checked the reference lists of relevant reviews for additional studies. After importing studies searched from databases into Endnote and excluding duplicate records, two authors (HY and XW or ZG) browsed titles and abstracts to initially determine potential eligible studies and then scanned full text to assess for final inclusion. Studies were included if they met all criteria: (1) they were historical or prospective cohort studies that simultaneously conducted full-cohort and sibling-comparison analyses; (2) they examined the association of caesarean delivery compared with vaginal delivery with offspring health outcomes; and (3) they reported relative risk (RR), odds ratio (OR), or hazard ratio (HR) with 95% confidence interval (CI). All searches and screening were independently conducted by two authors (HY and XW or ZG), and a third author resolved disagreements by discussion and adjudication.

### Data extraction and quality assessment

Two authors (HY and XW or ZG) independently extracted the following information from each study using a predetermined form: (1) first author and year of publication; (2) characteristics of the study, including study design, study location, study period, characteristics of the participants, sample size, groups of exposure, and outcome measures; and (3) effect estimates from both full-cohort and sibling-comparison analyses, including the number of participants, calculated effect size (e.g., OR, RR or HR [95% CI]), and details of adjustment for confounders. Whenever possible, we extracted the effect estimates that were most fully adjusted in the studies; if adjusted estimates were not available, unadjusted ones were extracted. If a study classified caesarean delivery into elective caesarean delivery and emergency caesarean delivery, we extracted all information on effect estimates. When needed, we contacted the original author for clarification.

Two reviewers (XW and ZG or HY) independently assessed the quality of the included studies according to the Newcastle–Ottawa Scale, which was developed to assess the risk of bias in observational studies including cohort studies [23]. Study group selection (4 stars), comparability between groups (2 stars), and outcome measure (3 stars) are considered in the scale for cohort study, with the maximum being 9 stars. We defined ≥ 7 stars as high quality, 4–6 as medium quality, and ≤ 3 as low quality. Two reviewers (XW and ZG or HY) independently extracted data and assessed the quality of the included studies, and any discrepancies were resolved by discussion with a third investigator.

### Data synthesis and statistical analysis

The primary analysis was to estimate the overall pooled ORs with the 95% CIs for caesarean delivery versus vaginal delivery on offspring health outcomes derived from full-cohort and sibling-comparison analyses separately. All adjusted effect sizes, including those for either elective or emergency caesarean delivery, were taken into account, implying that multiple effect sizes from the same studies may be included. Therefore, three-level meta-analytic models were used to pool the estimates to account for the dependence within studies, and the restricted maximum likelihood estimations were used to obtain the parameters [24]. Moreover, a comparative analysis was carried out to evaluate the justification for using three-level models, as opposed to ordinary two-level models.

Since adverse offspring health outcomes were rare [25, 26], we regarded HR and RR as approximate ORs [27]. Statistical heterogeneity was assessed using the *I*^*2*^ and* Q* statistic, and the sources of heterogeneity were explored by conducting subgroup analyses according to the type of caesarean delivery (elective caesarean delivery or emergency caesarean delivery), type of outcomes, method of adjustment for maternal age at delivery (without adjustment, adjusting as a categorical variable, or adjusting as a continuous variable). In the subgroup analysis concerning the type of caesarean delivery, two-level random-effects models based on the generic invariance method were used to pool the results as only one effect size in each study was included. To assess the robustness of the results, sensitivity analyses were made by serially excluding each study. Funnel plots and Begg’s rank correlation test were used to assess potential publication bias [28].

In the simulation study, we created a hypothetical cohort of over a million mother–child pairs with varying maternal ages at delivery based on the results of the meta-analysis (e.g., the overall pooled ORs of caesarean delivery on offspring health outcomes) and those from the literature (e.g., the prevalence of caesarean delivery). In this simulated cohort, approximately 20% of the children were siblings, while the remaining ones were independent observations. With the assumption that increasing maternal age at delivery is associated with a higher chance of caesarean delivery as well as a lower risk of adverse health outcomes of offspring [21, 29], the mode of delivery and the health outcome of each child were simulated. We performed both full-cohort and sibling-comparison analyses and compared the estimated effects at different levels of sibling similarity (i.e., correlation of maternal age at delivery among siblings) and for different methods of adjustment for maternal age at delivery (i.e., without adjustment, adjusting by 10-year age categories, adjusting by 5-year age categories, or adjusting as a continuous variable). Each scenario was simulated 100 times, after which the median and interquartile range over the 100 estimates were calculated. The simulations only focused on maternal age at delivery as the confounding factor, without considering any other potential confounders. Full details of the simulation study are provided in Additional file 2 [2, 21, 29–31].

Statistical analyses were performed using R software (version 4.2.2), and statistical tests were two-sided with a significance level of 0.05.

## Results

### Study characteristics

**Fig. 1:**
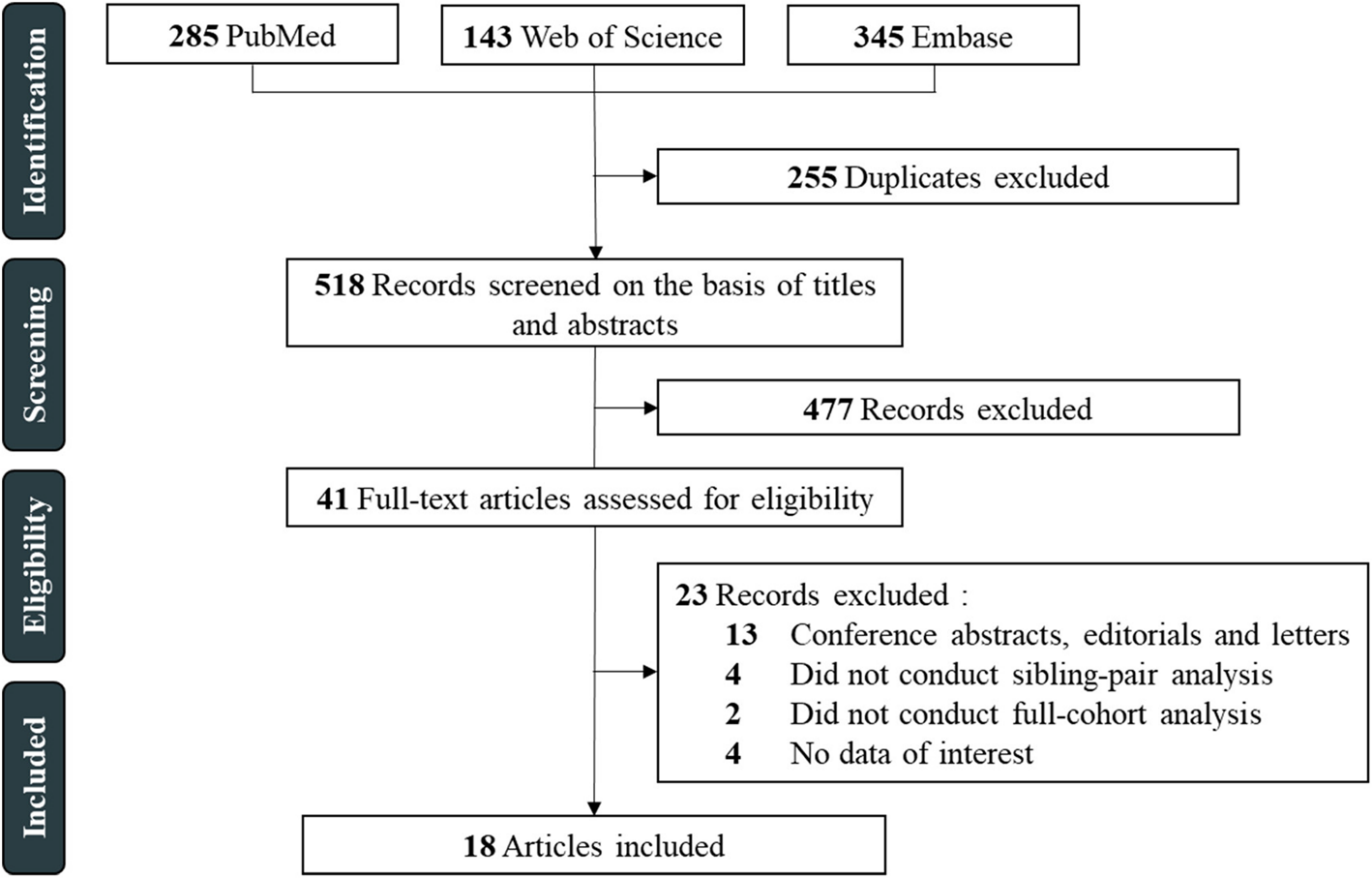
Flow diagram for study identification and selection

After scanning the titles, abstracts, or full texts, 18 studies involving 21,854,828 individuals were included in the meta-analysis (Fig. 1) [8, 12–16, 31–42]. Of these studies, 5 defined modes of delivery as either vaginal delivery or caesarean delivery, 5 categorized into unassisted vaginal delivery (reference group), assisted vaginal delivery (instrumental vaginal delivery), emergency caesarean delivery (intrapartum caesarean delivery), and elective caesarean delivery (prelabor caesarean delivery), 5 divided into vaginal delivery, elective caesarean delivery, and emergency caesarean delivery, and the remaining 3 studies divided into unassisted vaginal delivery, assisted vaginal delivery, and caesarean delivery. Two of the included studies presented two outcomes [15, 38], so a total of 31 estimates were involved in the primary analysis.

The included studies separately assessed the associations between caesarean delivery and 10 types of health outcomes. According to the International Classification of Diseases version 10, 9 studies focused on mental and behavioral disorders; 5 studies evaluated endocrine, nutritional and metabolic diseases; 2 studies concerned asthma; and the remaining 2 focused on multiple sclerosis and cardiorespiratory fitness, respectively. In terms of the effect estimates, 9 studies reported HRs of both full-cohort and sibling-comparison analyses [8, 12, 14, 34–37, 41, 42], 4 reported ORs [15, 31, 33, 38], 3 reported RRs [32, 39, 40], and the remaining 2 reported inconsistent types of effect size among full-cohort and sibling-comparison analyses [13, 16]. Regarding the adjustment for maternal age at delivery, 5 studies adjusted for it as a continuous variable [16, 31, 32, 36, 37], 11 adjusted for it as a categorical variable [8, 12, 14, 15, 33–35, 38, 40–42], and 2 studies did not adjust for it [13, 39]. The characteristics of the included studies are summarized in Table 1.

**Table 1:** Characteristics of the included studies

**Source**	**Study**			**Participants**	**Quality**	**Delivery modes**	**Outcome assessment**	**Full-cohort analyses**		**Sibling-comparison analyses**	
	**Design**	**Location**	**Period**					**No. of participants**	**Adjustment for confounders**	**No. of participants**	**Adjustment for confounders**
Ahlqvist et al. (2019) [32]	PCS	Sweden	1982–1987	Singleton pregnancy; no restrictions on gestational age at delivery.	7	Vaginal delivery; elective caesarean delivery; emergency caesarean delivery	Obesity	97,291	Prepregnancy BMI, maternal diabetes at delivery, maternal hypertension at delivery, maternal smoking, parity, parental education, maternal age, birth weight standardized according to gestational age, preeclampsia, gestational age	3346	Prepregnancy BMI, maternal diabetes at delivery, maternal hypertension at delivery, maternal smoking, parity, maternal age, birth weight standardized according to gestational age, preeclampsia, gestational age
Almqvist et al. (2012) [33]	PCS	Sweden	1993–1999	Singleton pregnancy; no restrictions on gestational age at delivery.	9	Unassisted vaginal delivery; assisted vaginal delivery; elective caesarean delivery; emergency caesarean delivery	Childhood asthma and allergic diseases	139,610	Gender, birth weight, gestational age, birth order, Apgar score, hypoxia/asphyxia, maternal age, smoking during pregnancy, mother living with father of the child, mother’s birth country, mother’s BMI	40,986	Gender, birth weight, gestational age, birth order, Apgar score, maternal age, smoking during pregnancy, mother living with father of the child, mother’s BMI
Axelsson et al. (2019) [34]	PCS	Denmark	1997–2010	Singleton pregnancy; no restrictions on gestational age at delivery.	9	Vaginal delivery; elective caesarean delivery; emergency caesarean delivery	Time to first autism diagnosis	671,606	Maternal age, parental age difference, parental education, maternal marital status, maternal smoking, gender, 5-min Apgar score, use of CPAP or a ventilator, asphyxia, parental epilepsy, preeclampsia or hypertension, gestational diabetes, parity, maternal antibiotic use during the pregnancy, maternal infections during the pregnancy, parental psychiatric history	7632	Maternal age, parental education, maternal marital status, maternal smoking, gender, 5-min Apgar score, use of CPAP or ventilator, asphyxia, preeclampsia or hypertension, gestational diabetes, parity, maternal antibiotics use during the pregnancy, maternal infections during the pregnancy
Axelsson et al. (2019) [8]	PCS	Denmark	1997–2010	Singleton pregnancy; no restrictions on gestational age at delivery.	9	Vaginal delivery; elective caesarean delivery; emergency caesarean delivery	ADHD	671,592	Maternal age, parental age difference, parental education, maternal marital status, maternal smoking, gender, 5-min Apgar score, instrument use at delivery, use of CPAP or ventilator, asphyxia, parental epilepsy, preeclampsia or hypertension, gestational diabetes, parity, induction of labor, induction of contractions, maternal antibiotics use during the pregnancy, maternal infections during the pregnancy, parental ADHD history	15,466	Maternal age, parental education, maternal marital status, maternal smoking, gender, 5-min Apgar score, instrument use at delivery, use of CPAP or ventilator, asphyxia, parental epilepsy, preeclampsia or hypertension, gestational diabetes, parity, induction of labor, induction of contractions, maternal antibiotics use during the pregnancy, maternal infections during the pregnancy
Axelsson et al. (2020) [35]	PCS	Denmark	1982–2001	Singleton pregnancy; no restrictions on gestational age at delivery.	9	Vaginal delivery; elective caesarean delivery; emergency caesarean delivery	Affective disorder	1,009,444	Gender, maternal age, paternal age difference, paternal education, maternal education, maternal marital status, parity, maternal psychiatric history, paternal psychiatric history	-	Gender, maternal age, paternal education, maternal education, maternal marital status, parity
Bråbäck et al. (2013) [15]	PCS	Sweden	1999–2006	Singleton pregnancy; only included term deliveries.	9	Unassisted vaginal delivery; assisted vaginal delivery; elective caesarean delivery; emergency caesarean delivery	Childhood asthma medication	199,837	Year of birth, gender, maternal and paternal asthma medication, socioeconomic indicators, maternal age, maternal smoking, urban/rural living, county, maternal history of diabetes and hypertension, premature rupture of the membranes, preeclampsia, gestational diabetes, gestational hypertension, maternal BMI, small for gestational age, large for gestational age, maternal fever during labor, chorioamnionitis, meconium aspiration, neonatal respiratory distress, transient tachypnoea	19,965	Gender, maternal age and parity, small for gestational age, large for gestational age, preeclampsia, maternal BMI, maternal diabetes, gestational diabetes, neonatal respiratory distress
Brander et al. (2016) [37]	PCS	Sweden	1973–1996	Singleton pregnancy; no restrictions on gestational age at delivery.	8	Unassisted vaginal delivery; assisted vaginal delivery; caesarean delivery	First instance of OCD diagnosis	2,386,686	Gender, year of birth, age of mother and father, parity	1,487,770	Gender, year of birth, age of mother and father, parity
Brander et al. (2018) [36]	PCS	Sweden	1973–2003	Singleton pregnancy; no restrictions on gestational age at delivery.	8	Unassisted vaginal delivery; assisted vaginal delivery; caesarean delivery	Tourette’s and chronic tic disorders	3,026,861	Gender, year of birth, age of mother and father, parity	1,895,884	Gender, year of birth, age of mother and father, parity
Curran et al. (2015) [14]	PCS	Sweden	1982–2010	Singleton pregnancy; no restrictions on gestational age at delivery.	8	Unassisted vaginal delivery; assisted vaginal delivery; elective caesarean delivery; emergency caesarean delivery	First diagnosis of autism spectrum disorder	2,697, 314	Year of birth, gender, maternal age, gestational age, 5-min Apgar score, maternal and paternal country of birth, small for gestational age, large for gestational age, first born, family income, maternal and paternal depression, bipolar disorder, nonaffective disorder	26,822	Year of birth, gender, maternal age, gestational age, 5-min Apgar score, paternal country of birth, small for gestational age, large for gestational age, first born, family income, maternal and paternal depression, bipolar disorder, nonaffective disorder
Curran et al. (2016) [12]	PCS	Sweden	1990–2008	Singleton pregnancy; no restrictions on gestational age at delivery.	9	Unassisted vaginal delivery; assisted vaginal delivery; elective caesarean delivery; emergency caesarean delivery	ADHD	1,722,548	Year of birth, gender, maternal age, maternal smoking during pregnancy, gestational age, 5-min Apgar score, maternal and paternal country of birth, small for gestational age, large for gestational age, firstborn, family income, maternal and paternal depression, bipolar disorder, non-affective disorder	17,382	Year of birth, gender, maternal age, maternal smoking during pregnancy, gestational age, 5-min Apgar score, paternal country of birth, small for gestational age, large for gestational age, firstborn, family income, maternal and paternal depression, bipolar disorder, non-affective disorder
Ekstrom et al. (2020) [31]	PCS	Sweden	1973–1987	Singleton pregnancy; no restrictions on gestational age at delivery.	8	Vaginal delivery; caesarean delivery	Low cardiorespiratory fitness	339,451	Birthweights standardized according to gestational age, gestational age, maternal age, parity, diabetes, hypertension, preeclampsia, SLE, parental education, household disposable income, parental country of birth, highest parental occupational class	20,590	Birthweights standardized according to gestational age, gestational age, maternal age, parity, diabetes, hypertension, preeclampsia, SLE, household disposable income, highest parental occupational class
Hawkins et al. (2019) [38]	PCS	United States	1980–2008	Singleton pregnancy and multiple pregnancy; no restrictions on gestational age at delivery.	9	Vaginal delivery; caesarean delivery	Childhood obesity	98,952	Gender, maternal race, maternal education, maternal age, marital status, number of children in household, year of birth	38,508	Gender, maternal education, maternal age, marital status, sibling order, year of birth
Khashan et al. (2014) [39]	PCS	Sweden	1982–2009	Singleton pregnancy; no restrictions on gestational age at delivery.	8	Unassisted vaginal delivery; assisted vaginal delivery; elective caesarean delivery; emergency caesarean delivery	Type 1 diabetes before the age of 15 years; type 1 diabetes; any diabetes diagnosis	2,638,083	Offspring age, year of birth, maternal diabetes, gestational age	12,174	Year of birth, maternal diabetes, gestational age
Li et al. (2022) [42]	PCS	Sweden	1973–2008	Singleton pregnancy; no restrictions on gestational age at delivery.	8	Unassisted vaginal delivery; assisted vaginal delivery; caesarean delivery	Stress-related disorders: PTSD, ASR, adjustment disorder, and other stress reactions	3,212,294	Paternal and maternal age, year of birth, gender, attained age, maternal country of birth, maternal education, history of parental psychiatric disorders	2,404,096	Paternal and maternal age, year of birth, gender, attained age
Martín-Calvo et al. (2020) [40]	PCS	Spain	1999–2016	Singleton pregnancy and multiple pregnancy; no restrictions on gestational age at delivery.	5	Vaginal delivery; caesarean delivery	Overweight or obesity	2791	Offspring’s age, gender, maternal age, maternal pregestational BMI, updated smoking habit, complications during pregnancy, gestational age, birth weight	341	Offspring’s age, gender, maternal age, maternal pregestational BMI, updated smoking habit, gestational age, birth weight
Nielsen et al. (2013) [13]	PCS	Denmark	1973–2005	Singleton pregnancy; no restrictions on gestational age at delivery.	8	Vaginal delivery; caesarean delivery	Offspring’s risk of multiple sclerosis	1,703,559	Birth order, gestational age, birth weight, maternal age, calendar period	1,980,226	Mother’s identity, maternal age, gender, birth weight, gestational age, birth order, birth cohort in 1-year intervals
Yuan et al. (2016) [16]	PCS	United States	1996–2012	Singleton pregnancy; no restrictions on gestational age at delivery.	7	Vaginal delivery; caesarean delivery	Obesity in offspring in childhood, adolescence, and early adulthood	22,068	Maternal age, race, region, year of birth, prepregnancy BMI, maternal height, gestational diabetes, preeclampsia, pregnancy-induced hypertension, gestational age at delivery, birth weight, prepregnancy smoking, previous caesarean delivery, gender, birth order	12,903	Maternal age, race, region, year of birth, prepregnancy BMI, maternal height, gestational diabetes, preeclampsia, pregnancy-induced hypertension, gestational age at delivery, birth weight, prepregnancy smoking, previous caesarean delivery, gender, birth order
Zhang et al. (2021) [41]	PCS	Sweden	1990–2003	Singleton pregnancy; only included term deliveries.	9	Vaginal delivery; elective caesarean delivery; emergency caesarean delivery	Neurodevelopmental disorders: ADHD, ASD, intellectual disability, and tic, communication, and learning disorders	1,179,341	Gender, year of birth, gestational age, paternal and maternal age, parity, mother education, maternal smoking during pregnancy, maternal and paternal history of psychiatric disorders, maternal hypertension, maternal diabetes, maternal infections during pregnancy, fetal malpresentation, large for gestational age, polyhydramnios, oligohydramnios, preeclampsia, pelvic disproportion, extra adjustment for placenta disorders, dystocia, failed induction, fetal distress, intrapartum caesarean delivery	808,020	Gender, year of birth, gestational age, paternal and maternal age, parity, mother education, maternal and paternal history of psychiatric disorders, maternal diabetes, maternal infections during pregnancy, large for gestational age, intrapartum caesarean delivery, further adjusted for dystocia, failed induction, fetal distress

*Abbreviations*: *PCS* Prospective cohort study, *ADHD* Attention deficit hyperactivity disorder, *ASD* Autism spectrum disorder, *ASR* Acute stress response, *BMI* Body mass index, *CPAP* Continuous positive airway pressure, *OCD* Obsessive–compulsive disorder, *PTSD* Posttraumatic stress disorder, *SLE* Systemic lupus erythematosus

### Quality assessment

Seventeen of the included studies were assessed to be high quality, and only one study was deemed to be medium quality [40]. Among 17 high-quality studies, 8 received 9 stars [8, 12, 15, 33–35, 38, 41], 7 received 8 stars [13, 14, 31, 36, 37, 39, 42], and 2 scored 7 stars [16, 32]. The detailed Newcastle–Ottawa scores of the included studies are shown in Additional file 3: Table S1.

### Associations between caesarean delivery and offspring health outcomes

**Fig. 2:**
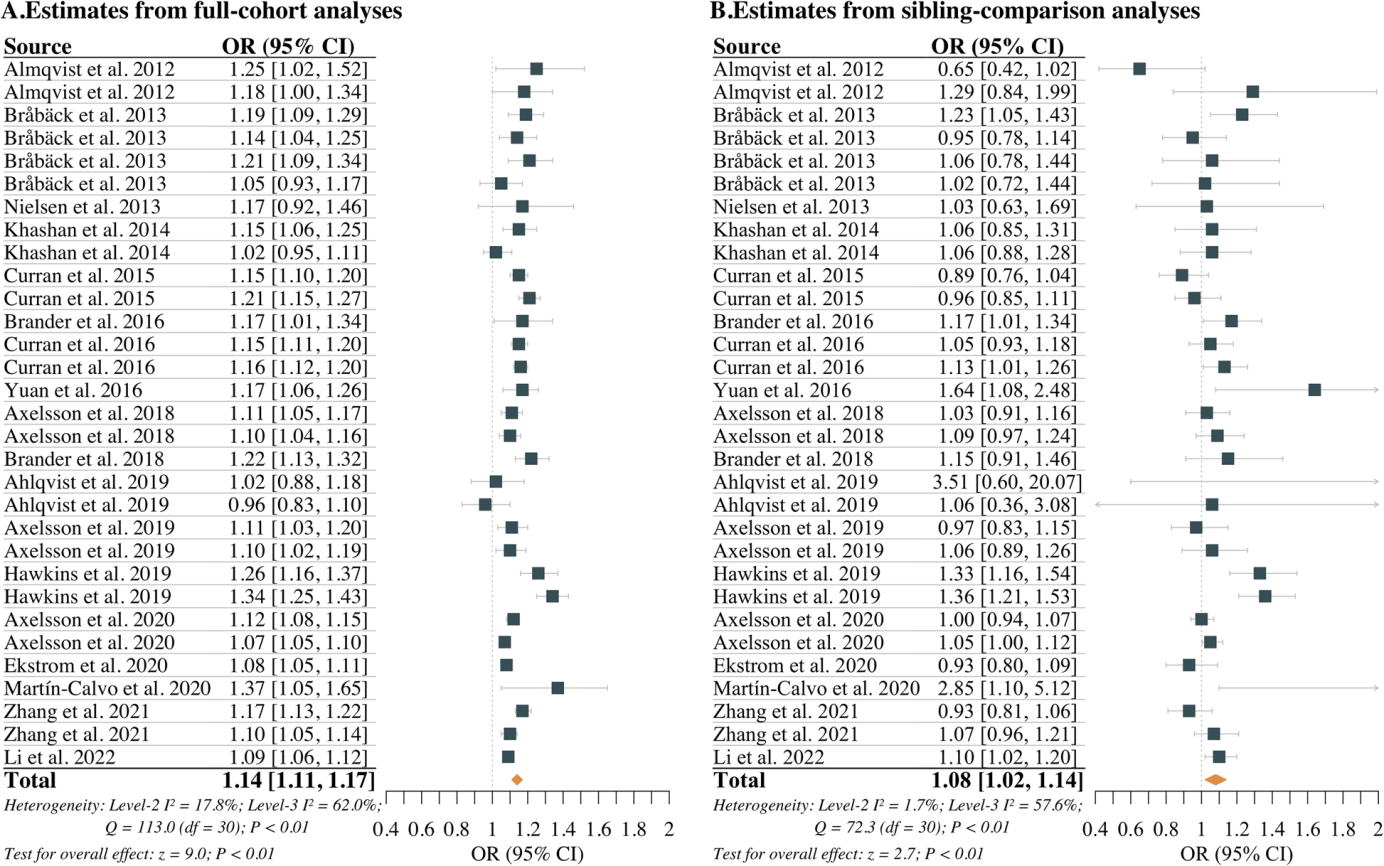
Caesarean delivery compared with vaginal delivery on offspring health outcomes

The three-level meta-analytic models revealed that caesarean delivery compared to vaginal delivery was significantly associated with increased risk of adverse offspring health outcomes. The pooling of effect estimates based on full-cohort analyses generated a summary OR of 1.14 (95% CI: 1.11 to 1.17), with 62.0% of the total variation attributed to between-study heterogeneity (level-3 *I*^*2*^ = 62.0%; *Q(df)* = 113.0(30); *P* < 0.01) (Fig. 2). Meanwhile, the pooled OR was significantly lower for estimates based on sibling-comparison analyses (*P* < 0.01), with a value of 1.08 (95% CI: 1.02 to 1.14) and 57.6% of the total variation attributed to between-study heterogeneity (*I*^*2*^ = 57.6%; *Q(df)* = 72.3(30); *P* < 0.01) (Fig. 2). The comparison between the three-level models and the two-level models showed that the former provided better fits (Additional file 3: Table S2).

### Subgroup analyses

Subgroup analyses were generally consistent with the primary analysis, with the pooled effect estimates of full-cohort analyses being relatively higher than those of sibling-comparison analyses (Table 2). When stratifying according to the type of caesarean delivery, the pooled ORs of elective caesarean delivery based on full-cohort and sibling-comparison analyses were 1.14 (95% CI: 1.13 to 1.16) and 1.01 (95% CI: 0.96 to 1.06), and those of emergency caesarean delivery were 1.10 (95% CI: 1.07 to 1.14) and 1.06 (95% CI: 1.02 to 1.10), respectively.

**Table 2:** Subgroup meta-analyses

**Subgroups**	**Number of estimates**	**Pooled OR for full-cohort analyses**	**Pooled OR for sibling-comparison analyses**
**Type of caesarean delivery**			
Elective caesarean delivery *vs.* vaginal delivery	11	1.14 [1.13, 1.16]	1.01 [0.96, 1.06]
Emergency caesarean delivery *vs.* vaginal delivery	11	1.10 [1.07, 1.14]	1.06 [1.02, 1.10]
**Health outcomes**			
Mental and behavioral disorders	15	1.13 [1.09, 1.18]	1.05 [1.00, 1.10]
Endocrine, nutritional and metabolic diseases	8	1.16 [1.09, 1.23]	1.27 [1.15, 1.41]
Asthma	6	1.17 [1.07, 1.29]	1.06 [0.93, 1.22]
Multiple sclerosis	1	1.17 [0.91, 1.52]	1.03 [0.62, 1.71]
Cardiorespiratory fitness	1	1.08 [0.96, 1.21]	0.93 [0.77, 1.12]
**Adjustment for maternal age at delivery**			
Did not adjust	3	1.10 [0.99, 1.22]	1.06 [0.85, 1.31]
Adjusted as a categorical variable	22	1.15 [1.11, 1.19]	1.07 [1.00, 1.15]
Adjusted as a continuous variable	6	1.12 [1.05, 1.19]	1.12 [0.98, 1.29]

*Abbreviation*: *OR* Odds ratio

When stratifying by the type of outcomes, the pooled ORs based on full-cohort versus sibling-comparison analyses for mental and behavioral disorders, asthma, multiple sclerosis, and low cardiorespiratory fitness were 1.13 (95% CI: 1.09 to 1.18) *vs.* 1.05 (95% CI: 1.00, 1.10), 1.17 (95% CI: 1.07 to 1.29) *vs.* 1.06 (95% CI: 0.93 to 1.22), 1.17 (95% CI: 0.91 to 1.52) *vs.* 1.03 (95% CI: 0.62 to 1.71), and 1.08 (0.96 to 1.21) *vs.* 0.93 (95% CI: 0.77 to 1.12), respectively. Nevertheless, in the subgroup of endocrine, nutritional and metabolic diseases, the pooled OR based on sibling-comparison analyses (1.27, 95% CI: 1.15 to 1.41) tended to be slightly higher than that based on full-cohort analyses (1.16, 95% CI: 1.09 to 1.23).

The discrepancies in the results between full-cohort and sibling-comparison analyses, as anticipated, appeared to vary with methods of adjustment for maternal age at delivery. Regarding the estimates that did not adjust for maternal age at delivery, the pooled OR based on full-cohort analyses was 1.10 (95% CI: 0.99 to 1.22), while that based on sibling-comparison analyses was 1.06 (95% CI: 0.85 to 1.31). In the estimates that adjusted for maternal age at delivery as a categorical variable, the pooled ORs of full-cohort and sibling-comparison analyses were 1.15 (95% CI: 1.11 to 1.19) and 1.07 (95% CI: 1.00 to 1.15), respectively. Notably, among the remaining estimates that adjusted for maternal age at delivery as a continuous variable, the pooled ORs based on full-cohort and sibling-comparison analyses were 1.12 (95% CI: 1.05 to 1.19) and 1.12 (95% CI: 0.98 to 1.29), respectively.

### Sensitivity analyses and assessment of publication bias

In the primary leave-1-out analyses, omitting any study did not significantly change the estimated effect size (Additional file 3: Table S3). The funnel plots suggested an absence of publication bias, whether based on full-cohort or sibling-comparison analyses (Additional file 4: Figure S1), and the Begg’s rank correlation test also did not indicate significant publication bias of the included studies (Additional file 3: Table S4).

### Simulations

**Fig. 3:**
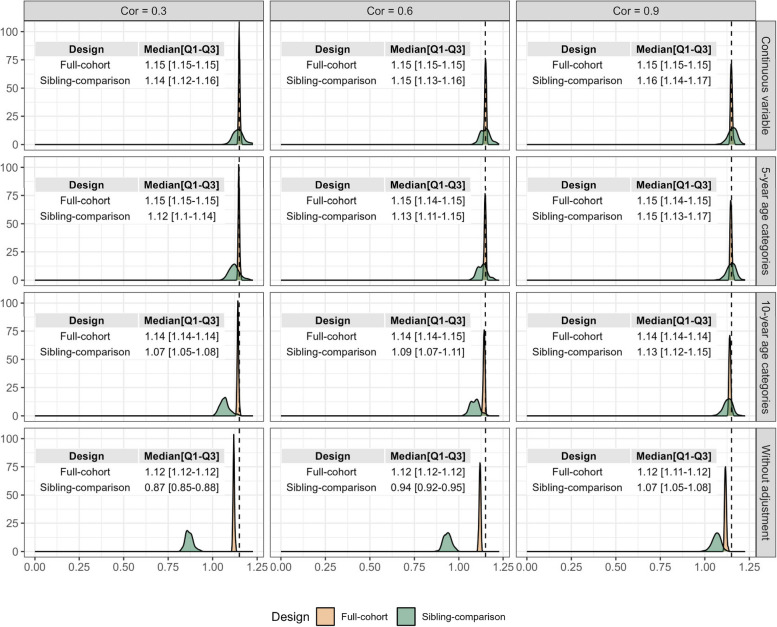
Distributions of estimates in the simulation study. The black dashed line indicates the “true effect” of caesarean delivery on offspring health that we set according to the results of our meta-analysis. “Cor” represents the correlation of maternal age at delivery between siblings

We simulated scenarios where insufficient adjustment for maternal age at delivery may lead to discrepancies between the results of full-cohort and sibling-comparison analyses. The distributions of the estimates derived from the two designs are shown in Fig. 3.

When siblings were less similar regarding maternal age at delivery (i.e., the correlation of maternal age at delivery between siblings was equal to 0.3), the difference between the estimates from the two designs increased as the adjustment became more insufficient. Specifically, when we adjusted maternal age at delivery as a continuous variable, the results from both designs were approximately equal to the true effect, while the estimates derived from full-cohort analyses were more concentrated. When we adjusted for maternal age at delivery as a categorical variable, the estimates from full-cohort analyses were still relatively close to the actual effect, while those from sibling-comparison analyses were far from the true value. As the similarity of maternal age at delivery increased, the difference between the results of the two designs decreased. For example, when we did not adjust for maternal age at delivery, the difference in the median of the estimates from the two designs changed from 0.25 to 0.05 as the correlation of maternal age at delivery between siblings changed from 0.3 to 0.9. In addition, we also found that regardless of whether conditional logistic regression or the between-within model was used in sibling-comparison analyses, the results of the simulation study were robust (Additional file 2).

## Discussion

### Principal findings

To our knowledge, this study is the first to synthesize and comprehensively investigate the associations of caesarean delivery with offspring health outcomes generated by full-cohort and sibling-comparison analyses. Given the high rate and potential adverse impacts of caesarean delivery, clarification of the seemingly contradictory evidence from these two types of analyses is of clinical and public health significance. As anticipated, the pooled OR of caesarean delivery with offspring health outcomes derived from sibling-comparison analyses was more conservative than that derived from full-cohort analyses. This phenomenon was more pronounced in the subgroup of studies that did not adjust for maternal age at delivery or adjusted for it as a categorical covariate.

Previous research has pointed out mathematically that the estimates from sibling-comparison design may be more biased when siblings are less similar regarding non-shared confounders [17]. In this study, we considered maternal age at delivery to be a main non-shared confounder for the following reasons. First, a vaginal delivery after previous caesarean is less frequent than a caesarean delivery after previous vaginal birth [18–20], so in sibling-comparison studies, children delivered by caesarean delivery were more likely to be born to older mothers. Therefore, the difference in maternal age at delivery between the two delivery modes in sibling-comparison studies is inherently larger than that in full-cohort studies. Meanwhile, maternal age at delivery is closely related to the health and well-being of offspring [43], since it relates to biological, social, economic, and behavioral factors that may affect a child’s development [44–46]. Older mothers generally have higher socioeconomic status and better parenting experience [47]. Thus, increasing maternal age might be associated with a lower risk of adverse health outcomes of offspring, which may in turn reduce the negative impacts of caesarean delivery on offspring health outcomes [48]. In addition, similar to many other continuous covariates, maternal age at delivery was often adjusted categorically in multivariate regression models. Adjustment for continuous confounders as categorical variables may inevitably result in residual confounding [49], and given the design nature, a sibling-comparison design compared to a full-cohort design would be particularly susceptible to such confounding [17]. Therefore, the effect estimates generated by sibling-comparison studies may be more likely to underestimate the underlying relationship between caesarean delivery and offspring health outcomes.

The simulation study further supported our hypothesis as well as findings from the meta-analysis. Simulated results demonstrated that the estimates from the full-cohort analyses were more concentrated, more accurate, and less affected by the inadequate adjustment of maternal age at delivery. In contrast, the estimates from the sibling-comparison analyses were dispersed and more susceptible to the influence of residual confounding. Notably, consistent with the findings in the meta-analysis, when we insufficiently adjusted for maternal age at delivery, the estimates of full-cohort analyses were always closer to the true value we set. Although fully adjusting confounders is far more complex than we simulated, we believe that the results of ordinary cohort studies with large sample sizes would be more accurate and robust than those of sibling-comparison studies, especially when the adjustment for non-shared confounders such as maternal age at delivery is inadequate.

Interestingly, we noticed that the effect of caesarean delivery on endocrine, nutritional and metabolic diseases, especially obesity or overweight, appeared to be overestimated, but not underestimated, in sibling-comparison analyses. A previous study found that when maternal age was greater than 30 years, it was associated with a higher risk of offspring being overweight or obese [50]. This may be due to the high prevalence of obesity among older women [51, 52], which may in turn negatively impact the development of the offspring’s metabolic system and ultimately result in metabolic diseases in offspring [53, 54]. Therefore, contrary to previous scenarios, older maternal age at delivery was positively associated with the outcome at this time, so sibling-comparison analyses compared to full-cohort analyses would be more likely to overestimate the effect size when the adjustment for maternal age at delivery was inadequate.

### Limitations of the study

This study has several limitations. First, multiple types of health outcomes, with potentially high heterogeneity, were included in the analyses. Although the subgroup analysis concerning different types of health outcomes was performed, the number of studies in some subgroups was limited. However, this meta-analysis did not focus on the effects of caesarean delivery on offspring health outcomes but rather on comparing the estimates of the effects from different designs. Second, the effect estimates of the included studies were inconsistent, including ORs, RRs, and HRs. We regarded both HRs and RRs as ORs to obtain a relatively conservative estimate. Third, due to the limited number of studies available, only maternal age at delivery was used as a proxy for similar inverse confounders. Future studies should investigate additional confounders to obtain a more comprehensive understanding of the associations. Fourth, the models we used in the simulation study may not perfectly reflect real-world scenarios. For instance, maternal age at delivery was considered as the only confounder, and the association of maternal age at delivery with offspring health outcomes was simply assumed to be linear. However, since the aim of the simulation study is to illustrate how inverse confounders such as maternal age at delivery may lead to the underestimation of sibling-comparison analyses, the discrepancy between the models and reality may not affect the results. Fifth, most included studies used data from Swedish or Danish national registers and might fail to be well-represented worldwide. Reassuringly, the results of these studies were proven to be consistent with those from other settings [55–57].

## Conclusions

The results of our meta-analysis and simulation study indicated that sibling-comparison analyses may underestimate the association of caesarean delivery with multiple offspring health outcomes due to inadequate adjustment of non-shared confounders such as maternal age at delivery. In contrast, full-cohort analyses provide more reliable estimates of this association. Therefore, it is advisable to future delivery-related studies to give priority to the large-sample cohort design. If using the sibling-comparison design, it is essential to carefully consider the impact of non-shared confounders and be cautious about the interpretation of the results.

### Supplementary Information

**Additional file 1.** Search Strategy.

**Additional file 2.** Details of Simulation Study.

**Additional file 3: Table S1.** Results of Quality Assessment. **Table S2.** Comparison Between Three-level Models and Two-level Models. **Table S3.** Results of Sensitivity Analyses. **Table S4.** Results of Begg’s Test.

**Additional file 4: Figure S1.** Funnel Plots.

## Data Availability

The raw data for the systematic review and meta-analysis is included in Table 1 and Fig. 2, and the details of the model used for the simulation study are included in Additional file 2.

## Abbreviations

ADHD: Attention deficit hyperactivity disorder
AIC: Akaike information criterion
ASD: Autism spectrum disorder
ASR: Acute stress response
BIC: Bayesian information criterion
BMI: Body mass index
CI: Confidence interval
CPAP: Continuous positive airway pressure
HR: Hazard ratio
OCD: Obsessive-compulsive disorder
OR: Odds ratio
PCS: Prospective cohort study
PRISMA: Preferred Reporting Items for Systematic Reviews and Meta-Analyses
PTSD: Posttraumatic stress disorder
RR: Relative risk
SLE: Systemic lupus erythematosus
VBAC: Vaginal birth after a previous caesarean
