# Supplementary material for: Association of caesarean delivery with offspring health outcomes in full-cohort versus sibling-comparison studies: a comparative meta-analysis and simulation study
Source: BMC Med. 2023 Sep 8;21:348. doi: 10.1186/s12916-023-03030-2 (PMC10486071; doi:10.1186/s12916-023-03030-2)
Supplement: Supplementary file 1 — Additional file 1. Search Strategy. [file 12916_2023_3030_MOESM1_ESM.docx]

**Search Strategy**

***Database: PubMed***

#1 "Delivery, Obstetric"[Mesh Terms]

#2 Cesarean[Text Word]

#3 Caesarean[Text Word]

#4 Abdominal Deliver*[Text Word]

#5 C-Section*[Text Word]

#6 Delivery Mode[Text Word]

#7 Mode of Delivery[Text Word]

#8 Delivery Method[Text Word] OR Delivery Methods[Text Word]

#9 Method of Delivery[Text Word] OR Methods of Delivery[Text Word]

#10 Delivery Type[Text Word]

#11 Type of Delivery[Text Word]

#12 Mode of Obstetric* Delivery[Text Word]

#13 #1 OR #2 OR #3 OR #4 OR #5 OR #6 OR #7 OR #8 OR #9 OR #10 OR #11 OR #12

#14 Sibling*[Text Word]

#15 Within-family[Text Word]

#16 Within-subject[Text Word]))

#17 Sister-pair[Text Word]

#18 Brother-pair[Text Word]

#19 #14 OR #15 OR #16 OR #17 OR #18

#20 "Cohort Studies"[Mesh Terms]

#21 Cohort*[Text Word]

#22 Prospective Study[Text Word]

#23 Longitudinal Study[Text Word]

#24 Concurrent Study[Text Word]

#25 Incidence Study[Text Word]

#26 Population-based*[Text Word]

#27 Follow-up[Text Word]

#28 #20 OR #21 OR #22 OR #23 OR #24 OR #25 OR #26 OR #27

#29 #13 AND #19 AND #28

#30 #29 NOT ((Systematic[Filter] OR Meta-Analysis[PT]) OR Review[PT])

***Database: Embase***

#1 'cesarean section'/exp OR 'cesarean section' OR 'c section*' OR 'c-section*' OR 'cesarean' OR 'caesarean'

#2 'obstetric delivery'/exp OR 'abdominal deliver*'

#3 'delivery type' OR 'type of delivery'

#4 'delivery method$' OR 'method$ of delivery'

#5 'delivery mode' OR 'mode of delivery'

#6 'mode of obstetric* delivery'

#7 #1 OR #2 OR #3 OR #4 OR #5 OR #6

#8 'sibling*'

#9 'within-family'

#10 'within-subject'

#11 'sister-pair'

#12 'brother-pair'

#13 #8 OR #9 OR #10 OR #11 OR #12

#14 'cohort analysis'/exp

#15 'cohort'

#16 'prospective study'

#17 'longitudinal study'

#18 'concurrent study'

#19 'incidence study'

#20 'population-based*'

#21 'follow up'

#22 'follow-up'

#23 #14 OR #15 OR #16 OR #17 OR #18 OR #19 OR #20 OR #21 OR #22

#24 #7 AND #13 AND #23

#25 #24 NOT ([cochrane review]/lim OR [systematic review]/lim OR [meta analysis]/lim)

***Database: Web of Science***

#1 TS = (“Cesarean Section” OR Cesarean OR Caesarean OR “Abdominal Deliver*” OR “C-Section$” OR “Delivery Mode” OR “Mode of Delivery” OR “Delivery Method$” OR “Method$ of Delivery” OR “Delivery Type” OR “Type of Delivery” OR “Mode of Obstetric* Delivery”)

#2 TS = (Sibling* OR “Within-family” OR “Within-subject” OR “Sister-pair” OR “Brother-pair”)

#3 TS = (Cohort OR “Prospective Study” OR “Longitudinal Study” OR “Concurrent Study” OR “Incidence Study” OR “Population-based*” OR “Follow-up”)

#4 #1 AND #2 AND #3
