# Supplementary material for: Association of caesarean delivery with offspring health outcomes in full-cohort versus sibling-comparison studies: a comparative meta-analysis and simulation study
Source: BMC Med. 2023 Sep 8;21:348. doi: 10.1186/s12916-023-03030-2 (PMC10486071; doi:10.1186/s12916-023-03030-2)
Supplement: Supplementary file 2 — Additional file 2. Details of Simulation Study. [file 12916_2023_3030_MOESM2_ESM.docx]

**Details of Simulation Study**

***Data generating model***

For simplicity, we assumed that all confounders other than maternal age at delivery are shared among siblings and that the causal effects of maternal age at delivery on all other variables are linear. Only the first and the second births of different delivery modes will be included, to make it easier to determine the interval of maternal age at delivery between the two groups.

The data generating model included equations (1) to (4) below, with (1) and (2) being the model that produced the data of independent mother-child pairs while (3) and (4) represent the model for siblings.

$X_{i}=\beta_{XA}A_{i}+\epsilon_{X_{i}}$ (1)

$\log\left( \frac{Y_{i}}{1-Y_{i}} \right)=\beta_{YA}A_{i}+\beta_{YX}X_{i}+\epsilon_{Y_{i}}$ (2)

$X_{ij}=\beta_{XA}^{*}A_{ij}+\epsilon_{X_{ij}}$ (3)

$\log\left( \frac{Y_{ij}}{1-Y_{ij}} \right)=\beta_{YA}A_{ij}+\beta_{YX}X_{ij}+\epsilon_{Y_{ij}}$ (4)

where $X_{i}$ and $Y_{i}$ represent the exposures (delivery mode) and outcomes of independent mother-child pairs respectively, while $X_{ij}$ and $Y_{ij}$ represent the corresponding variables of individual *j* in pair *i* among siblings. $A_{i}$ and $A_{ij}$ denote the maternal age at delivery. $\beta_{XA}$,$\beta_{XA}^{*}$, and$\beta_{YA}$ indicate the effect of maternal age at delivery on exposure among independent mother-child pairs, exposure among siblings, and outcome, respectively, and $\beta_{YX}$ represents the effect of delivery mode on outcome. $\epsilon_{X_{i}}$, $\epsilon_{Y_{i}}$, $\epsilon_{X_{ij}}$, and $\epsilon_{Y_{ij}}$ are random errors that follow a normal distribution. According to the results of our meta-analysis and previous literature, the difference in maternal age at delivery of the two delivery methods in siblings was set to approximately 2 to 3 times that of independent mother-child pairs [30, 31]; $\beta_{YX}$ was set to approximately 0.14 (close to the result of our meta-analysis), and the prevalence of caesarean delivery was 20% [2]. $\beta_{XA}$,$\beta_{XA}^{*}$ were positive, while $\beta_{YA}$ was negative [21, 29]. The basic relationship of the model is illustrated below.


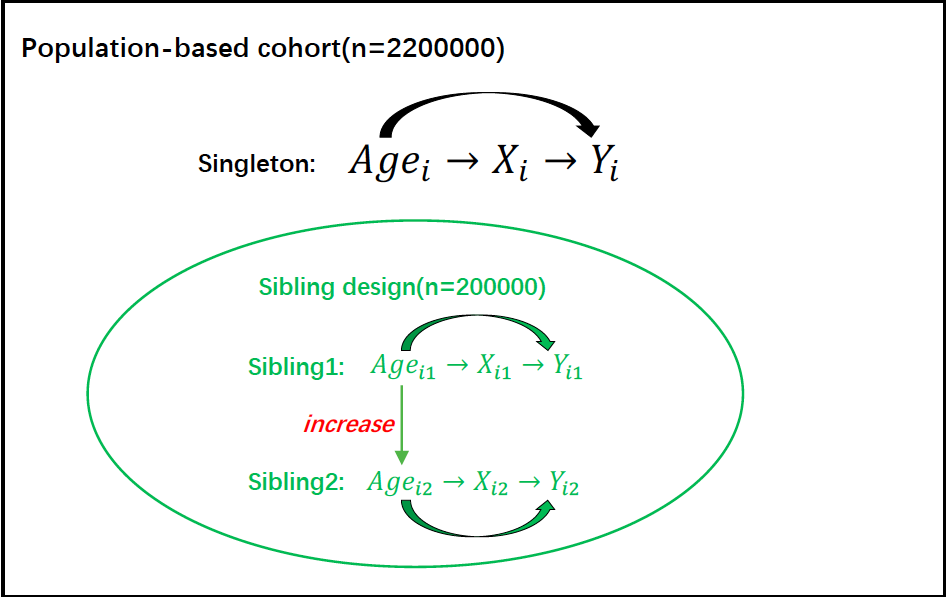


In consideration of the hierarchical nature of the data, we used between-within model and conditional logistic regression, the two most commonly used methods, for sibling-comparison analyses, while ordinary logistic model was used for full-cohort analyses. Simulation followed by estimation was done 100 times, after which the median of the 100 estimates was calculated.

***Distribution of maternal age at delivery***

The distributions of maternal age at delivery are shown below. As mentioned earlier, due to the higher rate of caesarean delivery in the second pregnancy, the difference in maternal age at delivery in sibling-comparison analyses is larger than that in full-cohort analyses.


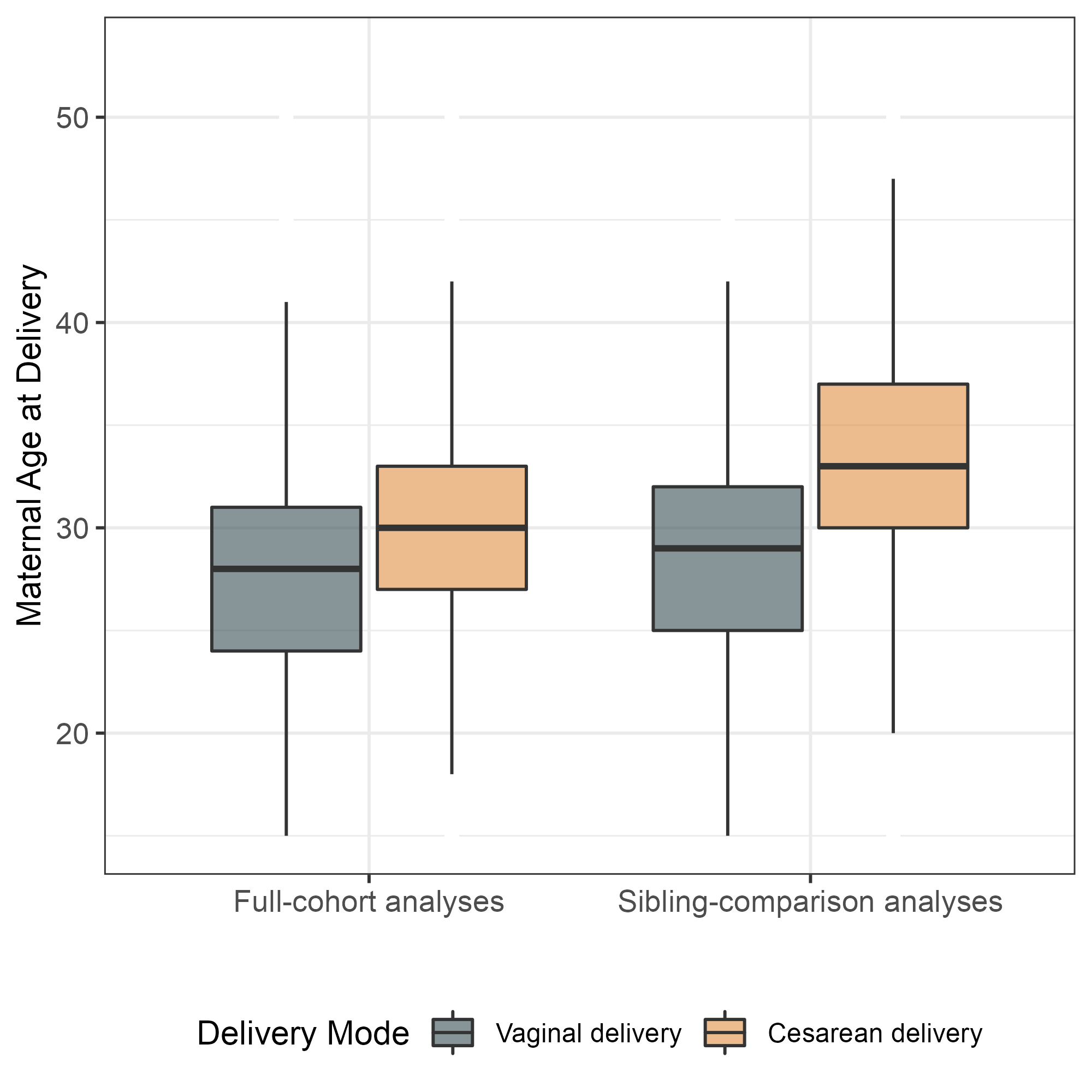


***Distributions of estimates***

Distributions of estimates derived from the two designs using three analysis methods are shown below. The black solid line indicates the “true effect” of caesarean delivery on offspring health that we set according to the results of our meta-analysis. Regardless of whether conditional logistic regression or between-within model was used in sibling-comparison analyses, the results of the simulation study were always similar to those described in the article.


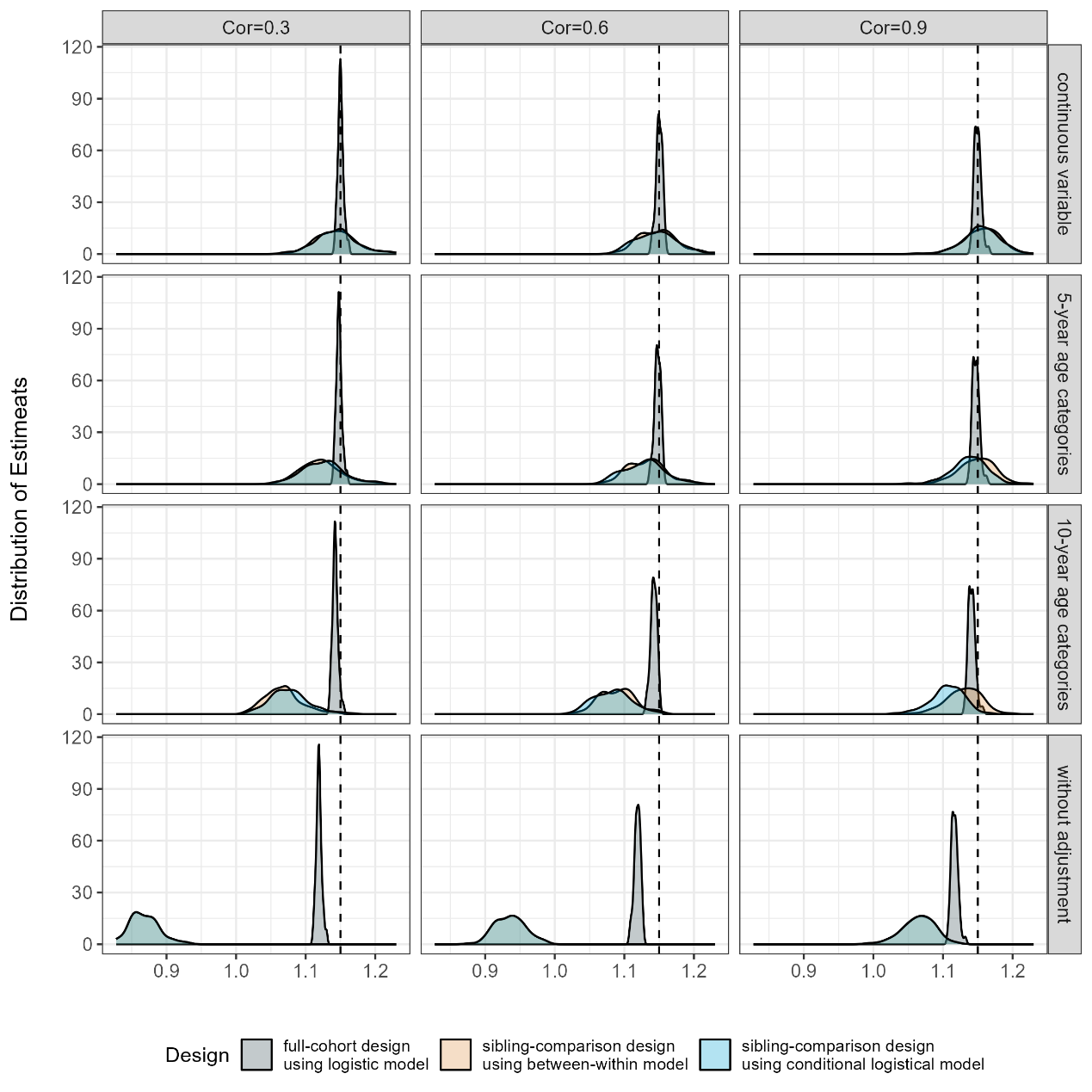


Notes: The black dashed line indicates the “true effect” of caesarean delivery on offspring health that we set according to the results of our meta-analysis. “Cor” represents the correlation of maternal age at delivery between siblings.
