# Supplementary material for: Association of caesarean delivery with offspring health outcomes in full-cohort versus sibling-comparison studies: a comparative meta-analysis and simulation study
Source: BMC Med. 2023 Sep 8;21:348. doi: 10.1186/s12916-023-03030-2 (PMC10486071; doi:10.1186/s12916-023-03030-2)
Supplement: Supplementary file 3 — Additional file 3: Table S1. Results of Quality Assessment. Table S2. Comparison Between Three-level Models and Two-level Models. Table S3. Results of Sensitivity Analyses. Table S4. Results of Begg’s Test. [file 12916_2023_3030_MOESM3_ESM.docx]

**Additional file 3**

**Table S1.** Results of Quality Assessment

**Table S2.** Comparison Between Three-level Models and Two-level Models

**Table S3.** Results of Sensitivity Analyses

**Table S4.** Results of Begg’s Test

**Table S1. Results of Quality Assessment**

| **Almqvist et al. 2012** | | | | | | | |
| --- | --- | --- | --- | --- | --- | --- | --- |
| **Category** | **Item** | | **Description** | | | | **Stars** |
| Selection | Representativeness of the exposed cohort | | A population-based follow-up study based on the Swedish national registers contains maternal and child characteristics data on > 98% of all births in Sweden. | | | | ⭐ |
|  | Selection of the non-exposed cohort | | The cohort was limited to the first two siblings of mothers who had given birth to those two children within the allocated time frame. | | | | ⭐ |
|  | Ascertainment of exposure | | Information on mode of delivery in the Medical Birth Register. | | | | ⭐ |
|  | Demonstration that outcome of interest was not present at start of study | | Yes (Outcome: asthma and allergic diseases). | | | | ⭐ |
| Comparability | Comparability of cohorts on the basis of the design or analysis | | Adjusted for the child characteristics gender, birth order, birth weight, gestational age and Apgar score at 5 min, maternal age, smoking during pregnancy, mother living with father of the child, mother’s birth country and mother’s BMI. The paired analyses were adjusted for the same variables as the cohort analyses. | | | | ⭐⭐ |
| Outcome | Assessment of outcome | | Asthma outcome variables were collected from national health registers as diagnosis or asthma medication (ICD-10 J45-J46; ATC code R03). | | | | ⭐ |
|  | Was follow-up long enough for outcomes to occur | | Yes (During the 13th year of life for children born June 1993–May 1996 and during the 10th year of life for those born form June 1996 to June 1999). | | | | ⭐ |
|  | Adequacy of follow up of cohorts | | Complete follow up. | | | | ⭐ |
| **Bråbäck et al. 2013** | | | | | | | |
| **Category** | | **Item** | **Description** | | **Stars** | | |
| Selection | | Representativeness of the exposed cohort | The catchment population for this study were all full term (gestational week 37-<42), singleton children born in Sweden between January 1999 and December 2006, according to the Swedish Medical Birth Register. | | ⭐ | | |
|  |  | Selection of the non-exposed cohort | The non-exposed cohort were drawn from the same dataset as the exposed cohort. | | ⭐ | | |
|  |  | Ascertainment of exposure | Information about mode of delivery was collected from Swedish Medical Birth Register. | | ⭐ | | |
|  |  | Demonstration that outcome of interest was not present at start of study | Yes (Outcome: dispensed inhaled corticosteroid (ICS), a marker of asthma). | | ⭐ | | |
| Comparability | | Comparability of cohorts on the basis of the design or analysis | Model 1 was adjusted for year of birth and sex only, in Model 2 it added other socio-demographic confounders (including smoking and maternal age) and parental dispensed ICS, in Model 3 it added perinatal risk factors associated with mode of delivery. | | ⭐⭐ | | |
| Outcome | | Assessment of outcome | This study created three proxy indicators for asthma based on data of retrieved prescriptions from the Swedish Prescribed Drug Register. | | ⭐ | | |
|  |  | Was follow-up long enough for outcomes to occur | Yes (Aged 2-5 and 6-9 years). | | ⭐ | | |
|  |  | Adequacy of follow up of cohorts | Complete follow up. | | ⭐ | | |
| **Nielsen et al. 2013** | | | | | | | |
| **Category** | | **Item** | **Description** | | **Stars** | | |
| Selection | | Representativeness of the exposed cohort | The study identified all singleton births occurring between January 1973 and December 2005 based on the data from the Danish Civil Registration System (CRS). | | ⭐ | | |
|  |  | Selection of the non-exposed cohort | The non-exposed cohort were drawn from the same dataset as the exposed cohort. | | ⭐ | | |
|  |  | Ascertainment of exposure | The Danish Medical Birth Register has data on all the live births and stillbirths by women with permanent residence in Denmark and contains detailed information about each childbirth, including mode of delivery (vaginal birth versus cesarean delivery). | | ⭐ | | |
|  |  | Demonstration that outcome of interest was not present at start of study | Yes (Outcome: multiple sclerosis (MS)). | | ⭐ | | |
| Comparability | | Comparability of cohorts on the basis of the design or analysis | The following variables were included as covariates in the Poisson regression model: birth weight, gestational age and birth order. | | ⭐⭐ | | |
| Outcome | | Assessment of outcome | The study identified the MS cases in the study cohort in the Danish Multiple Sclerosis Register, comprising information on all cases of MS in Denmark since 1956. | | ⭐ | | |
|  |  | Was follow-up long enough for outcomes to occur | Yes. Each cohort member was followed from the date of birth or 1 January 1977, whichever came later, until the study end on 31 December 2007, the person’s death, emigration or their diagnosis/first symptoms of MS, whichever came first. | | ⭐ | | |
|  |  | Adequacy of follow up of cohorts | No statement (Censoring). | |  | | |
| **Khashan et al. 2014** | | | | | | | |
| **Category** | | **Item** | **Description** | | **Stars** | | |
| Selection | | Representativeness of the exposed cohort | Using data from the Swedish Medical Birth Register, the study identified almost all children born in Sweden between January 1982, and December 2009. The Swedish Medical Birth Register contains obstetric, maternal, and neonatal data on > 99% of births in Sweden. | | ⭐ | | |
|  |  | Selection of the non-exposed cohort | The non-exposed cohort were drawn from the same dataset as the exposed cohort. | | ⭐ | | |
|  |  | Ascertainment of exposure | Data on obstetric complications were retrieved from the Medical Birth Register. Mode of delivery was classified into unassisted vaginal delivery, instrumental vaginal delivery (forceps or vacuum extraction), emergency caesarean delivery and elective caesarean delivery. | | ⭐ | | |
|  |  | Demonstration that outcome of interest was not present at start of study | Yes (Outcome: childhood type 1 diabetes (T1D), before 15 years of age). | | ⭐ | | |
| Comparability | | Comparability of cohorts on the basis of the design or analysis | The Poisson model was adjusted for offspring age as a time-dependent variable, year of birth, gestational age, and maternal pre-pregnancy diabetes. For sibling control analyses, the final conditional logistic models were adjusted for year of birth, maternal prepregnancy diabetes, and gestational age. | | ⭐⭐ | | |
| Outcome | | Assessment of outcome | The Swedish National Patient Register contains records of inpatient diagnoses in Sweden since 1964 (full national coverage since 1987) and outpatient diagnoses since 2001. | | ⭐ | | |
|  |  | Was follow-up long enough for outcomes to occur | The cohort was followed from the date of birth until the onset of the outcome measure, 15th birthday (for the primary outcome only), death, migration, or 31 December 2009 (end of the study period). | | ⭐ | | |
|  |  | Adequacy of follow up of cohorts | No statement (Censoring). | |  | | |
| **Curran et al. 2015** | | | | | | | |
| **Category** | | **Item** | **Description** | | **Stars** | | |
| Selection | | Representativeness of the exposed cohort | The study cohort consisted of all singleton live births in Sweden from January 1982, through December 2010, using data from the Swedish Medical Birth Register, the Swedish National Patient Register, and the Swedish Multi-Generation Register. More than 99% of all births in Sweden are recorded in the Swedish Medical Birth Register. | | ⭐ | | |
|  |  | Selection of the non-exposed cohort | The non-exposed cohort were drawn from the same dataset as the exposed cohort. | | ⭐ | | |
|  |  | Ascertainment of exposure | Although the Swedish Medical Birth Register began in 1973, variables indicating emergency and elective caesarean delivery are available from 1982, which marks the beginning of the investigation. | | ⭐ | | |
|  |  | Demonstration that outcome of interest was not present at start of study | Yes (Outcome: autism spectrum disorder (ASD)). | | ⭐ | | |
| Comparability | | Comparability of cohorts on the basis of the design or analysis | In fully adjusted models, the study stratified by every fourth birth year and adjusted for year of birth, infant sex, maternal age, gestational age, maternal and paternal citizenship, small for gestational age, large for gestational age, 5-minute Apgar score, parity, social welfare status, family disposable income, and maternal and paternal depression, bipolar disorder, and nonaffective disorder in the total population. | | ⭐⭐ | | |
| Outcome | | Assessment of outcome | Information on ASD status and date of first diagnosis was obtained from the Swedish National Patient Register. | | ⭐ | | |
|  |  | Was follow-up long enough for outcomes to occur | Children were followed up until first diagnosis of ASD, death, migration, or 31 December 2011 (end of study period), whichever came first. | | ⭐ | | |
|  |  | Adequacy of follow up of cohorts | No statement (Censoring). | |  | | |
| **Brander et al. 2016** | | | | | | | |
| **Category** | | **Item** | **Description** | | **Stars** | | |
| Selection | | Representativeness of the exposed cohort | The study cohort, consisting of all 2,421,284 live singleton births in Sweden from January 1973, through December 1996. | | ⭐ | | |
|  |  | Selection of the non-exposed cohort | The non-exposed cohort were drawn from the same dataset as the exposed cohort. | | ⭐ | | |
|  |  | Ascertainment of exposure | Information about all prenatal exposures was retrieved from the Swedish Medical Birth Register. Obstetric delivery was divided into 3 hierarchical categories: cesarean section, assisted vaginal delivery (use of forceps or vacuum extraction), and unassisted vaginal delivery. | | ⭐ | | |
|  |  | Demonstration that outcome of interest was not present at start of study | Yes (The first instance of a recorded obsessive-compulsive disorder (OCD) diagnosis in the National Patient Register constituted the outcome). | | ⭐ | | |
| Comparability | | Comparability of cohorts on the basis of the design or analysis | The analysis was initially adjusted for sex and year of birth, and all measured confounders including parity, paternal age and maternal age at childbirth were adjusted for in the fully adjusted model. | | ⭐⭐ | | |
| Outcome | | Assessment of outcome | The first instance of a recorded OCD diagnosis in the National Patient Register constituted the outcome. | | ⭐ | | |
|  |  | Was follow-up long enough for outcomes to occur | Participants were followed up until first diagnosis of OCD, migration, death, or end of follow-up (13 December 2013), whichever came first. | | ⭐ | | |
|  |  | Adequacy of follow up of cohorts | No statement (Censoring). | |  | | |
| **Curran et al. 2016** | | | | | | | |
| **Category** | | **Item** | **Description** | | **Stars** | | |
| Selection | | Representativeness of the exposed cohort | The study cohort consisted of all singleton live births in Sweden from 1990 to 2008 using data from Swedish national registers. Over 99% of all births in Sweden are recorded in the Medical Birth Register. | | ⭐ | | |
|  |  | Selection of the non-exposed cohort | The non-exposed cohort were drawn from the same dataset as the exposed cohort. | | ⭐ | | |
|  |  | Ascertainment of exposure | Though the Medical Birth Register began in 1973, variables indicating emergency and elective caesarean delivery are only available from 1982. | | ⭐ | | |
|  |  | Demonstration that outcome of interest was not present at start of study | Yes (Outcome: attention-deficit/hyperactivity disorder (ADHD)). | | ⭐ | | |
| Comparability | | Comparability of cohorts on the basis of the design or analysis | All models in the study partially adjusted for year of birth. In fully adjusted models, it also included infant gender, maternal age, gestational age, maternal and paternal citizenship, small for gestational age (SGA), LGA (large for gestational age infant), 5-min Apgar score, parity, maternal smoking during pregnancy, social welfare status, parental education, family disposable income and maternal and paternal depression, bipolar disorder and non-affective disorder. | | ⭐⭐ | | |
| Outcome | | Assessment of outcome | Inpatient International Classification of Diseases version 10 (ICD-10) codes are available beginning in 1997, and outpatient available from 2001. Prescriptions are available beginning in July, 2005. | | ⭐ | | |
|  |  | Was follow-up long enough for outcomes to occur | The study began follow-up of participants on their third birthday for any child born after 1 January 1994. As ICD-10 was available beginning in 1997, for children who turned 3 years before 1997 we began follow-up on 1 January 1997. Participants were followed until ADHD diagnosis or medication, death, emigration or the end of study period (31 December 2011). | | ⭐ | | |
|  |  | Adequacy of follow up of cohorts | Subjects lost to follow up unlikely to introduce bias and description provided of those lost. | | ⭐ | | |
| **Yuan et al. 2016** | | | | | | | |
| **Category** | | **Item** | **Description** | | **Stars** | | |
| Selection | | Representativeness of the exposed cohort | The Growing Up Today Study (GUTS) is an ongoing prospective cohort study of young adults followed up since 1 September 1996. | |  | | |
|  |  | Selection of the non-exposed cohort | The non-exposed cohort were drawn from the same dataset as the exposed cohort. | | ⭐ | | |
|  |  | Ascertainment of exposure | Mode of delivery (cesarean vs vaginal) was reported by the participants’ mothers in 2009 using a questionnaire aimed at collecting lifetime pregnancy information. | | ⭐ | | |
|  |  | Demonstration that outcome of interest was not present at start of study | Yes (Outcome: obesity). | | ⭐ | | |
| Comparability | | Comparability of cohorts on the basis of the design or analysis | The multivariable adjusted models included terms for maternal age at delivery, race, region, year of birth, prepregnancy BMI, maternal height, gestational diabetes, preeclampsia, pregnancy-induced hypertension, gestational age at delivery, birth weight, prepregnancy smoking, previous cesarean delivery, offspring sex, and birth order. | | ⭐⭐ | | |
| Outcome | | Assessment of outcome | In each follow-up questionnaire, participants reported their height and weight, which are validly reported by preadolescents, adolescents, and adults, although there is potential misclassification of obesity based on self-reported anthropometry. | |  | | |
|  |  | Was follow-up long enough for outcomes to occur | followed up via questionnaire from ages 9 to 14 through ages 20 to 28 years. | | ⭐ | | |
|  |  | Adequacy of follow up of cohorts | Complete follow up. | | ⭐ | | |
| **Brander et al. 2018** | | | | | | | |
| **Category** | | **Item** | **Description** | | **Stars** | | |
| Selection | | Representativeness of the exposed cohort | The study cohort consisted of all live singleton births in Sweden from January 1973 to December 2003. | | ⭐ | | |
|  |  | Selection of the non-exposed cohort | The non-exposed cohort were drawn from the same dataset as the exposed cohort. | | ⭐ | | |
|  |  | Ascertainment of exposure | Information about all perinatal exposures was retrieved from the Medical Birth Register (MBR). In this study, unless otherwise specified, data from 1973 until 2003 were employed. | | ⭐ | | |
|  |  | Demonstration that outcome of interest was not present at start of study | Yes (Outcome: Tourette’s disorder (TD) and chronic tic disorders (CTD)). | | ⭐ | | |
| Comparability | | Comparability of cohorts on the basis of the design or analysis | This study adjusted for sex and year of birth and in the fully adjusted model, it adjusted for all measured confounders, including parity and maternal age at childbirth) and paternal age at childbirth. | | ⭐⭐ | | |
| Outcome | | Assessment of outcome | The first instance of a recorded TD or CTD diagnosis in the Swedish National Patient Register (NPR) constituted the outcome using the classification of the International Classification of Diseases (ICD) in its 8th (ICD-8 code 306.2), 9th (ICD-9 code 307C) and 10th versions (ICD-10 codes F95.0 (transient tic disorder), F95.1 (chronic motor or vocal tic disorder), F95.2 (TD), F95.8 (other tic disorders) or F95.9 (unspecified tic disorder)). | | ⭐ | | |
|  |  | Was follow-up long enough for outcomes to occur | The cohort was followed from birth until first diagnosis of TD/CTD, emigration, death or end of follow-up (31 December 2013), whichever came first. | | ⭐ | | |
|  |  | Adequacy of follow up of cohorts | No statement (Censoring). | |  | | |
| **Ahlqvist et al. 2019** | | | | | | | |
| **Category** | | **Item** | | **Description** | | **Stars** | |
| Selection | | Representativeness of the exposed cohort | | Using the Swedish Medical Birth Register (MBR), which contains validated birth data on approximately 99% of the Swedish population, all male singletons born between 1982 and 1987 available in the MBR were sampled. | | ⭐ | |
|  |  | Selection of the non-exposed cohort | | The non-exposed cohort were drawn from the same dataset as the exposed cohort. | | ⭐ | |
|  |  | Ascertainment of exposure | | Using the Swedish MBR, the study obtained information on recorded mode of delivery (vaginal or cesarean delivery), which was supplemented with information on indication for cesarean delivery, which yielded the primary trichotomized exposure coded as (1) vaginal delivery, (2) elective caesarean delivery, and (3) nonelective caesarean delivery. | | ⭐ | |
|  |  | Demonstration that outcome of interest was not present at start of study | | Yes (Outcome: obesity among young adult male offspring). | | ⭐ | |
| Comparability | | Comparability of cohorts on the basis of the design or analysis | | The study adjusted for prepregnancy maternal BMI, maternal diabetes at delivery, maternal hypertension at delivery, self-reported maternal smoking at the commencement of pregnancy, parity, birth weight in grams standardized according to week of gestational age using the total population as reference, preeclampsia, gestational age, maternal age at delivery and the highest level of paternal and maternal education. | | ⭐ | |
| Outcome | | Assessment of outcome | | At conscription, weight and height were measured and transformed to World  Health Organization categories of body mass index (BMI). | | ⭐ | |
|  |  | Was follow-up long enough for outcomes to occur | | A cohort of 97,291 males born between 1982 and 1987 were followed from birth until conscription (median 18 years of age) if they conscripted before 2006. | |  | |
|  |  | Adequacy of follow up of cohorts | | All analyses were conducted as complete-case analyses because we a priori hypothesized any missing data to be missing completely at random and potentially missing not at random in a few cases. | | ⭐ | |
| **Axelsson et al. 2019** | | | | | | | |
| **Category** | | **Item** | **Description** | | **Stars** | | |
| Selection | | Representativeness of the exposed cohort | This population-based, prospective cohort study linked nationwide registers of data for native Danish singleton live births in Denmark from 1997 to 2010 and 671,592 Danish singleton children, not diagnosed with ADHD and still alive and living in Denmark on  their second birthday were included. | | ⭐ | | |
|  |  | Selection of the non-exposed cohort | The non-exposed cohort were drawn from the same dataset as the exposed cohort. | | ⭐ | | |
|  |  | Ascertainment of exposure | Data about the mode of delivery were obtained from the Medical Birth Registry from 1973 to 2010. | | ⭐ | | |
|  |  | Demonstration that outcome of interest was not present at start of study | Yes (Outcome: attention deficit hyperactivity disorder (ADHD)). | | ⭐ | | |
| Comparability | | Comparability of cohorts on the basis of the design or analysis | In the fully adjusted model, childhood antibiotics use, mode of delivery, maternal age at birth, parental age difference, parental education, maternal marital status, maternal smoking, infant sex, 5-minute Apgar score, instrument use at delivery, use of continuous positive airway pressure (CPAP) or ventilator, asphyxia, parental epilepsy, preeclampsia or hypertension, gestational diabetes, parity, induction of labor, induction of contractions, maternal antibiotics use during the pregnancy, maternal infections during the pregnancy and parental ADHD history were adjusted. | | ⭐⭐ | | |
| Outcome | | Assessment of outcome | ADHD case, defined as the assignment of an ADHD diagnosis, attention deficit disorder (ADD) diagnosis, or at least two redeemed prescriptions for ADHD/ADD medication on separate dates. Both patients admitted to the hospital and those treated in an outpatient hospital clinic were included, as were primary and secondary discharge diagnoses. | | ⭐ | | |
|  |  | Was follow-up long enough for outcomes to occur | The children were censored at time of death, emigration, the International Classification of Diseases version 10 (ICD-10) diagnosis of an Organic Mental Disorder, Mental Disorders due to Substance Abuse or Psychotic Disorders, or end of follow-up by 31 December 2014, whichever occurred first. | | ⭐ | | |
|  |  | Adequacy of follow up of cohorts | Subjects lost to follow up unlikely to introduce bias and description provided of those lost. | | ⭐ | | |
| **Axelsson et al. 2019** | | | | | | | |
| **Category** | | **Item** | **Description** | | **Stars** | | |
| Selection | | Representativeness of the exposed cohort | This is a population-based, prospective cohort study and all live-born children born in Denmark to Danish parents between January 1997 and December 2010 were identified. | | ⭐ | | |
|  |  | Selection of the non-exposed cohort | The non-exposed cohort were drawn from the same dataset as the exposed cohort. | | ⭐ | | |
|  |  | Ascertainment of exposure | Cesarean deliveries were performed either prelabor (elective) or intrapartum (after onset of labor). The study used vaginal delivery as the reference category. | | ⭐ | | |
|  |  | Demonstration that outcome of interest was not present at start of study | Yes (Outcome: autism). | | ⭐ | | |
| Comparability | | Comparability of cohorts on the basis of the design or analysis | In the fully adjusted model, childhood antibiotics use, mode of delivery, maternal age at birth, parental age difference, parental education, maternal marital status, maternal smoking, infant sex, 5-minute Apgar score, instrument use at delivery, use of continuous positive airway pressure (CPAP) or ventilator, asphyxia, parental epilepsy, preeclampsia or hypertension, gestational diabetes, parity, induction of labor, induction of contractions, maternal antibiotics use during the pregnancy, maternal infections during the pregnancy and parental attention deficit hyperactivity disorder (ADHD) history were adjusted. | | ⭐⭐ | | |
| Outcome | | Assessment of outcome | The outcome was time to first autism diagnosis, defined as the assignment of an autism spectrum disorder diagnosis. Autism spectrum disorder diagnoses in the Danish Psychiatric Central Research Register have been validated previously, and 97% of cases were confirmed by reviewing medical records. | | ⭐ | | |
|  |  | Was follow-up long enough for outcomes to occur | We censored children at time of death, emigration, ICD10 diagnoses of organic mental disorders, mental disorders because of substance abuse, schizophrenia, schizotypal or delusional disorders, or the end of the follow-up period on 31 December 2014, whichever occurred first. | | ⭐ | | |
|  |  | Adequacy of follow up of cohorts | Subjects lost to follow up unlikely to introduce bias and description provided of those lost. | | ⭐ | | |
| **Hawkins et al. 2019** | | | | | | | |
| **Category** | | **Item** | **Description** | | **Stars** | | |
| Selection | | Representativeness of the exposed cohort | This study used data from the Linked the Collecting Electronic Nutrition Trajectory Data Using e-Records of Youth (CENTURY) Study, a longitudinal clinical database created through the linkage of well-child visits with each child’s Massachusetts birth certificate, containing data on 306,147 children from 1980 to 2008. | | ⭐ | | |
|  |  | Selection of the non-exposed cohort | The non-exposed cohort were drawn from the same dataset as the exposed cohort. | | ⭐ | | |
|  |  | Ascertainment of exposure | The hospital also recorded the mode of delivery, which we dichotomized into caesarean versus no. | | ⭐ | | |
|  |  | Demonstration that outcome of interest was not present at start of study | Yes (Outcome: childhood obesity). | | ⭐ | | |
| Comparability | | Comparability of cohorts on the basis of the design or analysis | Model was adjusted for the presence of multiple siblings per family and included the following covariates: child sex, maternal race/ethnicity, maternal education, maternal age, marital status, number of children in household, and child year of birth. The second model included only siblings (≥2 children per family) and was adjusted for the same covariates, including clustering on the family identifier | | ⭐⭐ | | |
| Outcome | | Assessment of outcome | We extracted height and weight data from electronic health records collected at well-child visits. Medical assistants measured height and weight according to the written protocol of Atrius Health-affiliated health centers. | | ⭐ | | |
|  |  | Was follow-up long enough for outcomes to occur | Obesity outcomes at age 2 included children who were ≥2 and <3 years of age and outcomes at age 5 included children who were ≥ 5 and <6 years of age. | | ⭐ | | |
|  |  | Adequacy of follow up of cohorts | Subjects lost to follow up unlikely to introduce bias. | | ⭐ | | |
| **Axelsson et al. 2020** | | | | | | | |
| **Category** | | **Item** | | **Description** | **Stars** | | |
| Selection | | Representativeness of the exposed cohort | | Initially, all children born alive in Denmark between January 1982 and December 2001 were identified. The study then included the 1,009,444 Danish children who were singleton births. | ⭐ | | |
|  |  | Selection of the non-exposed cohort | | The non-exposed cohort were drawn from the same dataset as the exposed cohort. | ⭐ | | |
|  |  | Ascertainment of exposure | | The mode of delivery exposure variable was separated into three categories: pre-labor CS, intrapartum CS, and the reference category of vaginal delivery. | ⭐ | | |
|  |  | Demonstration that outcome of interest was not present at start of study | | Yes (Outcome: affective disorders). | ⭐ | | |
| Comparability | | Comparability of cohorts on the basis of the design or analysis | | Observed potential confounding variables for the effects of mode of delivery on affective disorders were adjusted progressively at five nested adjustment levels: mode of delivery, offspring sex, maternal age at birth, parental education, marital status and parity. For the standard Cox model, paternal age difference, and parental psychiatric history were also adjusted for. | ⭐⭐ | | |
| Outcome | | Assessment of outcome | | The primary outcome was any episode of an affective disorder, based on either a diagnosis or a redeemed prescription. Diagnoses were selected according to the International Classification of Disease version 10 (ICD-10). The study used the classification codes DF30–DF33 and DF38.00. | ⭐ | | |
|  |  | Was follow-up long enough for outcomes to occur | | These children were followed from 30 days after their 13th birthday for a total of 8,880,794 person years until an affective disorder was diagnosed or censoring at death,  emigration, diagnosis of a higher-ranking psychiatric disorder (ICD being hierarchical), or until the end of the follow-up period on 31 December 2014. | ⭐ | | |
|  |  | Adequacy of follow up of cohorts | | Subjects lost to follow up unlikely to introduce bias and description provided of those lost. | ⭐ | | |
| **Ekstrom et al. 2020** | | | | | | | |
| **Category** | | **Item** | | **Description** | **Stars** | | |
| Selection | | Representativeness of the exposed cohort | | It is a population-based longitudinal cohort study using nationwide register linked data on Swedish males born between 1973–1987. The resulting cohort (N = 613,489, 80.5%) was matched with conscription data as recorded in the Swedish Military Service Conscription Register. | ⭐ | | |
|  |  | Selection of the non-exposed cohort | | The non-exposed cohort were drawn from the same dataset as the exposed cohort. | ⭐ | | |
|  |  | Ascertainment of exposure | | The Swedish Medical Birth Register (MBR), containing compulsory reported data from all birth clinics on nearly all deliveries in Sweden since 1973, was used to collect data on modes of delivery, birth characteristics and maternal medical histories. | ⭐ | | |
|  |  | Demonstration that outcome of interest was not present at start of study | | No (Outcome: Watt-maximum (Wmax), a validated proxy measure of cardiorespiratory fitness (CRF), was defined as outcome). |  | | |
| Comparability | | Comparability of cohorts on the basis of the design or analysis | | We adjusted for birthweight and gestational age since both are positive predictors of CRF later in life. Maternal covariates at the time of delivery included age, parity, diabetes, hypertension, preeclampsia during pregnancy and systemic lupus erythematosus. Within-family analyses were adjusted similarly as other analyses, with the exception of parental education and parental country of birth which did not vary between brothers. | ⭐⭐ | | |
| Outcome | | Assessment of outcome | | All recruits called for conscription in the eighteenth life-year, who were considered medically fit, were invited to take the adjustable resistance cycle ergometer test during standardized nationwide conscription. The maximum work output achieved by each recruit at exhaustion, measured in Watts, was recorded as their respective Wmax. | ⭐ | | |
|  |  | Was follow-up long enough for outcomes to occur | | The analytic sample was followed until December 31, 2005. | ⭐ | | |
|  |  | Adequacy of follow up of cohorts | | Complete follow up. | ⭐ | | |
| **Martín-Calvo et al. 2020** | | | | | | | |
| **Category** | | **Item** | **Description** | | **Stars** | | |
| Selection | | Representativeness of the exposed cohort | Women in the “Seguimiento Universidad de Navarra” cohort starting from 1999 were provided structured information regarding their pregnancy history and their children’s health through online cross-sectional questionnaires. No major differences in baseline characteristics existed between women who did or did not agree to participate. | |  | | |
|  |  | Selection of the non-exposed cohort | The non-exposed cohort were drawn from the same dataset as the exposed cohort. | | ⭐ | | |
|  |  | Ascertainment of exposure | Women completed the first questionnaire (Q1) for each pregnancy during the follow-up in SUN. Q1 included questions about type of delivery (caesarean or vaginal). | |  | | |
|  |  | Demonstration that outcome of interest was not present at start of study | Yes (Outcome: overweight). | | ⭐ | | |
| Comparability | | Comparability of cohorts on the basis of the design or analysis | Multivariable models were adjusted for offspring’s sex and age, mother’s age, prepregnancy BMI, updated smoking habit, complications during pregnancy, gestational age and birth weight. In further analyses, the study additionally adjusted for breast feeding and administration of antibiotics during labor. | | ⭐⭐ | | |
| Outcome | | Assessment of outcome | Q2 included retrospective questions about current weight and height. The main outcome was the risk of overweight or obesity. | |  | | |
|  |  | Was follow-up long enough for outcomes to occur | Participants completed two brief questionnaires sent by email between March and June 2017. | |  | | |
|  |  | Adequacy of follow up of cohorts | Complete follow up. | | ⭐ | | |
| **Zhang et al. 2021** | | | | | | | |
| **Category** | | **Item** | **Description** | | **Stars** | | |
| Selection | | Representativeness of the exposed cohort | This Swedish register-based cohort study included 1,179,341 term-birth singletons born between January 1990, and December 2003. All individuals were linked to their full siblings, maternal and paternal half siblings, and maternal full cousins. | | ⭐ | | |
|  |  | Selection of the non-exposed cohort | The non-exposed cohort were drawn from the same dataset as the exposed cohort. | | ⭐ | | |
|  |  | Ascertainment of exposure | Information on mode of delivery has been recorded in the Medical Birth Register | | ⭐ | | |
|  |  | Demonstration that outcome of interest was not present at start of study | Yes (Outcome: any neurodevelopmental disorder). | | ⭐ | | |
| Comparability | | Comparability of cohorts on the basis of the design or analysis | Adjusted for child’s sex and year of birth, gestational age, age of mother and father at birth, parity, mother’s highest education level at birth, maternal smoking during pregnancy, and maternal and paternal history of psychiatric disorders, maternal hypertension, maternal diabetes, maternal infections during pregnancy, fetal malpresentation, large for gestational age, polyhydramnios, oligohydramnios, preeclampsia and pelvic disproportion and extra adjustment for placenta disorders, dystocia, failed induction, and fetal distress for intrapartum CD. | | ⭐⭐ | | |
| Outcome | | Assessment of outcome | This study identified cases using the first instance of a recorded International Classification of Disease version 9 (ICD-9) or version 10 (ICD-10) diagnosis in the National Patient Register. | | ⭐ | | |
|  |  | Was follow-up long enough for outcomes to occur | 1,179,341 individuals were followed up from birth until the first diagnosis (differing between the outcomes), emigration, death, or December 31, 2013, whichever came first. For some outcomes (i.e., psychiatric disorders) that tend to have a later onset, this study trimmed the main cohort to births between 1 January 1990, and 31 December 1997, to allow for longer follow-ups. | | ⭐ | | |
|  |  | Adequacy of follow up of cohorts | Complete follow up. | | ⭐ | | |
| **Li et al. 2022** | | | | | | | |
| **Category** | | **Item** | **Description** | | **Stars** | | |
| Selection | | Representativeness of the exposed cohort | Since 1973, the Swedish Medical Birth Register (MBR) has collected information on >98% of deliveries in Sweden, starting from women’s first visit to prenatal care, through delivery and birth care. Based on the MBR, 3,546,149 singleton live births from January 1973 to December 2008 were identified. | | ⭐ | | |
|  |  | Selection of the non-exposed cohort | The non-exposed cohort were drawn from the same dataset as the exposed cohort. | | ⭐ | | |
|  |  | Ascertainment of exposure | Mode of delivery was categorized as cesarean section, instrumental vaginal delivery (forceps or vacuum extraction), and non-instrumental vaginal delivery. | | ⭐ | | |
|  |  | Demonstration that outcome of interest was not present at start of study | Because stress-related disorders are usually not diagnosed in individuals below 5 years of age, this study followed all individuals from their 5th birthday. | | ⭐ | | |
| Comparability | | Comparability of cohorts on the basis of the design or analysis | The study adjusted for offspring sex, attained age, maternal country of birth, maternal educational level, and history of parental psychiatric disorders. | | ⭐ | | |
| Outcome | | Assessment of outcome | This study identified any first inpatient hospital or outpatient specialist visit with a stress-related disorder as the main diagnosis, using the codes of the 8–10th Swedish versions of International Classification of Disease. | | ⭐ | | |
|  |  | Was follow-up long enough for outcomes to occur | During the long-term follow-up up to 35 years (median 17.4 years), we identified 55,511 individuals diagnosed with stress-related disorders for the population analysis and 37,433 for the sibling analysis | | ⭐ | | |
|  |  | Adequacy of follow up of cohorts | The follow-up was censored at first emigration, death or December 31st, 2013, whichever came first. | | ⭐ | | |

**Table S2.** **Comparison Between Three-level Models and Two-level Models**

| Model | AIC | BIC | Likelihood ratio test | |
| --- | --- | --- | --- | --- |
|  |  |  | χ^2^ | *P* value |
| **Full-cohort analyses** | | | | |
| Three-level model | -73.269 | -69.065 | 3.923 | 0.047 |
| Two-level model | -71.346 | -68.543 |  |  |
| **Sibling-comparison analyses** | | | | |
| Three-level model | -19.89 | -15.68 | 4.312 | 0.038 |
| Two-level model | -17.58 | -14.77 |  |  |

Abbreviations: AIC, Akaike information criterion; BIC, Bayesian information criterion.

**Table S3. Results of Sensitivity Analyses**

| **Excluded study** | **Pooled odds ratio (95% confidence interval)** | |
| --- | --- | --- |
|  | **Full-cohort analyses** | **Sibling-comparison analyses** |
| Almqvist et al. 2012 | 1.14 [1.10, 1.17] | 1.08 [1.02, 1.14] |
| Bråbäck et al. 2013 | 1.14 [1.10, 1.17] | 1.08 [1.02, 1.14] |
| Nielsen et al. 2013 | 1.14 [1.11, 1.17] | 1.08 [1.02, 1.14] |
| Khashan et al. 2014 | 1.14 [1.11, 1.18] | 1.08 [1.02, 1.14] |
| Curran et al. 2015 | 1.14 [1.10, 1.17] | 1.09 [1.03, 1.15] |
| Brander et al. 2016 | 1.14 [1.11, 1.17] | 1.07 [1.01, 1.13] |
| Curran et al. 2016 | 1.14 [1.10, 1.17] | 1.08 [1.02, 1.14] |
| Yuan et al. 2016 | 1.14 [1.10, 1.17] | 1.07 [1.02, 1.13] |
| Brander et al. 2018 | 1.14 [1.10, 1.17] | 1.08 [1.02, 1.14] |
| Ahlqvist et al. 2019 | 1.15 [1.11, 1.18] | 1.08 [1.02, 1.14] |
| Axelsson et al. 2018 | 1.14 [1.11, 1.18] | 1.08 [1.02, 1.15] |
| Axelsson et al. 2019 | 1.14 [1.11, 1.18] | 1.08 [1.02, 1.15] |
| Hawkins et al. 2019 | 1.13 [1.10, 1.15] | 1.05 [1.01, 1.08] |
| Axelsson et al. 2020 | 1.14 [1.11, 1.18] | 1.08 [1.02, 1.15] |
| Ekstrom et al. 2020 | 1.14 [1.11, 1.18] | 1.09 [1.03, 1.15] |
| Martín-Calvo et al. 2020 | 1.14 [1.11, 1.17] | 1.07 [1.02, 1.13] |
| Zhang et al. 2021 | 1.14 [1.11, 1.18] | 1.09 [1.02, 1.15] |
| Li et al. 2022 | 1.14 [1.11, 1.18] | 1.08 [1.02, 1.14] |

**Table S4. Results of Begg’s Test**

| **Type of design** | **z-score** | ***P* value^α^** |
| --- | --- | --- |
| Full-cohort analyses | 0.63 | 0.529 |
| Sibling-cohort analyses | 0.76 | 0.444 |

^α^ *P* value greater than 0.05 indicated no significant publication bias.
