# Supplementary material for: Association of caesarean delivery with offspring health outcomes in full-cohort versus sibling-comparison studies: a comparative meta-analysis and simulation study
Source: BMC Med. 2023 Sep 8;21:348. doi: 10.1186/s12916-023-03030-2 (PMC10486071; doi:10.1186/s12916-023-03030-2)

**Additional file 4**

**Figure S1. Funnel Plots**

**A.** Funnel plot for estimates from full-cohort analyses

**
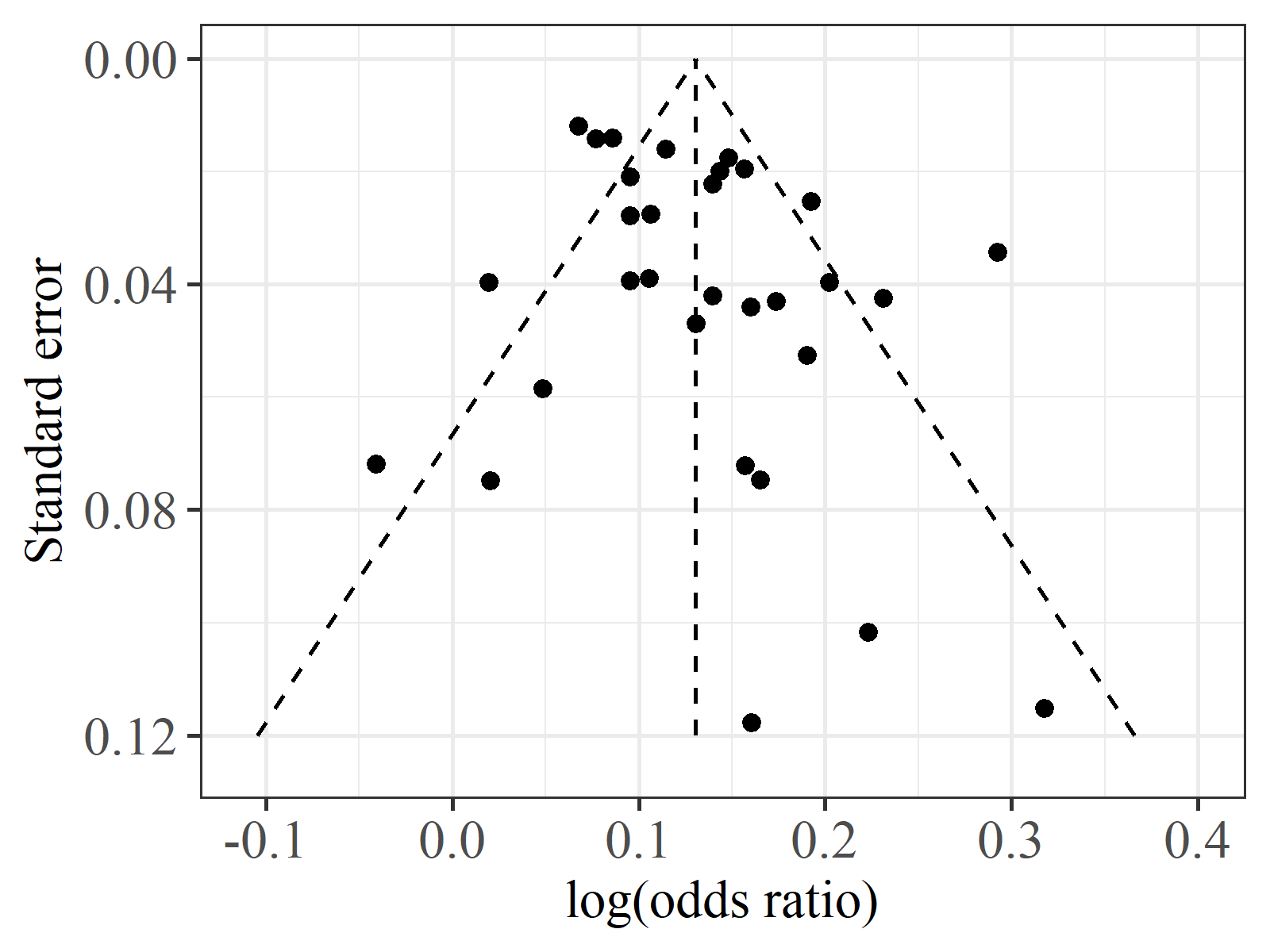
**

**B.** Funnel plot for estimates from sibling-comparison analyses


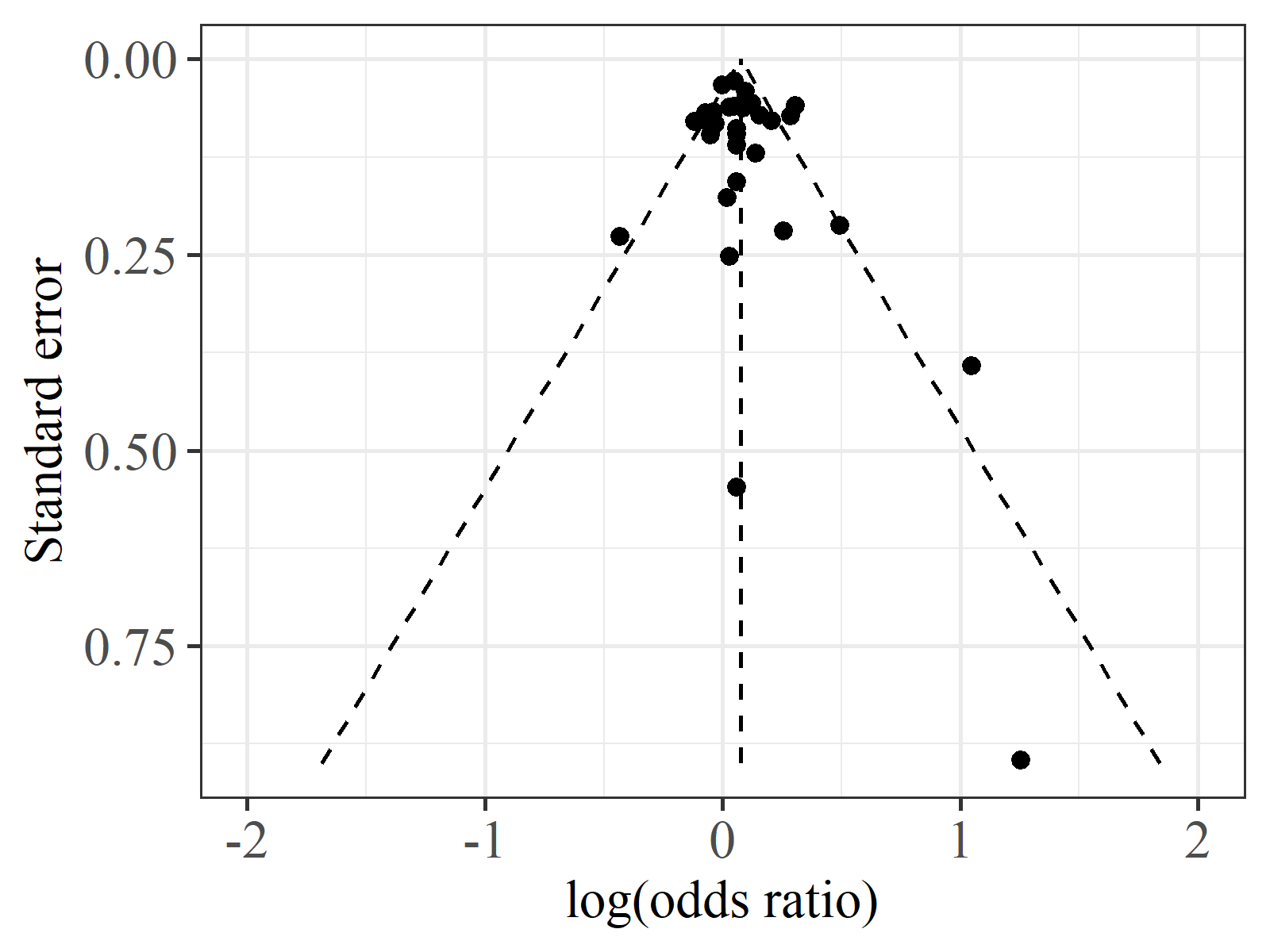

Supplement: Supplementary file 4 — Additional file 4: Figure S1. Funnel Plots. [file 12916_2023_3030_MOESM4_ESM.docx]
